# Bayesian modeling of spatially differentiated multivariate enamel defects of the children’s primary maxillary central incisor teeth

**DOI:** 10.1186/s12874-024-02211-8

**Published:** 2024-04-15

**Authors:** Everette P. Keller, Andrew B. Lawson, Carol L. Wagner, Susan G. Reed

**Affiliations:** 1 Department of Public Health Sciences, College of Medicine, Medical University of South Carolina, Charleston, SC USA; 2 Department of Pediatrics, College of Medicine, Medical University of South Carolina, Charleston, SC USA; 3 School of Medicine, Usher Institute, University of Edinburgh, Edinburgh, UK

**Keywords:** Bayesian, MCMC, Gibbs variable selection, Multivariate, Dental defects, Opacity, Hypoplasia, Post eruptive breakdown, Dental caries

## Abstract

**Background:**

The analysis of dental caries has been a major focus of recent work on modeling dental defect data. While a dental caries focus is of major importance in dental research, the examination of developmental defects which could also contribute at an early stage of dental caries formation, is also of potential interest. This paper proposes a set of methods which address the appearance of different combinations of defects across different tooth regions. In our modeling we assess the linkages between tooth region development and both the type of defect and associations with etiological predictors of the defects which could be influential at different times during the tooth crown development.

**Methods:**

We develop different hierarchical model formulations under the Bayesian paradigm to assess exposures during primary central incisor (PMCI) tooth development and PMCI defects. We evaluate the Bayesian hierarchical models under various simulation scenarios to compare their performance with both simulated dental defect data and real data from a motivating application.

**Results:**

The proposed model provides inference on identifying a subset of etiological predictors of an individual defect accounting for the correlation between tooth regions and on identifying a subset of etiological predictors for the joint effect of defects. Furthermore, the model provides inference on the correlation between the regions of the teeth as well as between the joint effect of the developmental enamel defects and dental caries. Simulation results show that the proposed model consistently yields steady inferences in identifying etiological biomarkers associated with the outcome of localized developmental enamel defects and dental caries under varying simulation scenarios as deemed by small mean square error (MSE) when comparing the simulation results to real application results.

**Conclusion:**

We evaluate the proposed model under varying simulation scenarios to develop a model for multivariate dental defects and dental caries assuming a flexible covariance structure that can handle regional and joint effects. The proposed model shed new light on methods for capturing inclusive predictors in different multivariate joint models under the same covariance structure and provides a natural extension to a nested hierarchical model.

**Supplementary Information:**

The online version contains supplementary material available at 10.1186/s12874-024-02211-8.

## Background

**Fig. 1:**
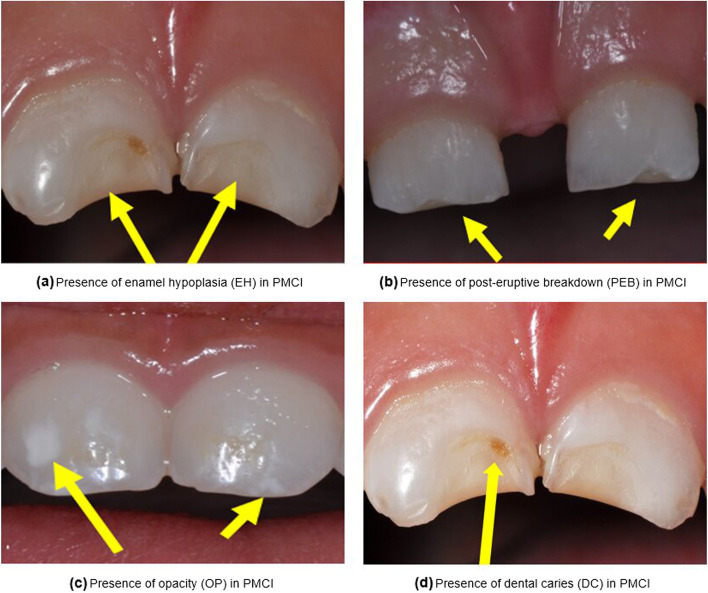
Panels displaying type of localized defect or dental caries in PMCI teeth as indicated by the yellow arrows. Panel (**a**) identifies the presence of enamel hypoplasia (EH) indicated by a lesser amount of enamel. Panel (**b**) shows the presence of post-eruptive breakdown (PEB) identified by the enamel wearing away. Panel (**c**) displays the presence of opacity (OP) identified by a whiter (or yellower) enamel color. Panel (**d**) shows the presence of dental caries (DC) identified by the spot of decay in the enamel

**Fig. 2:**
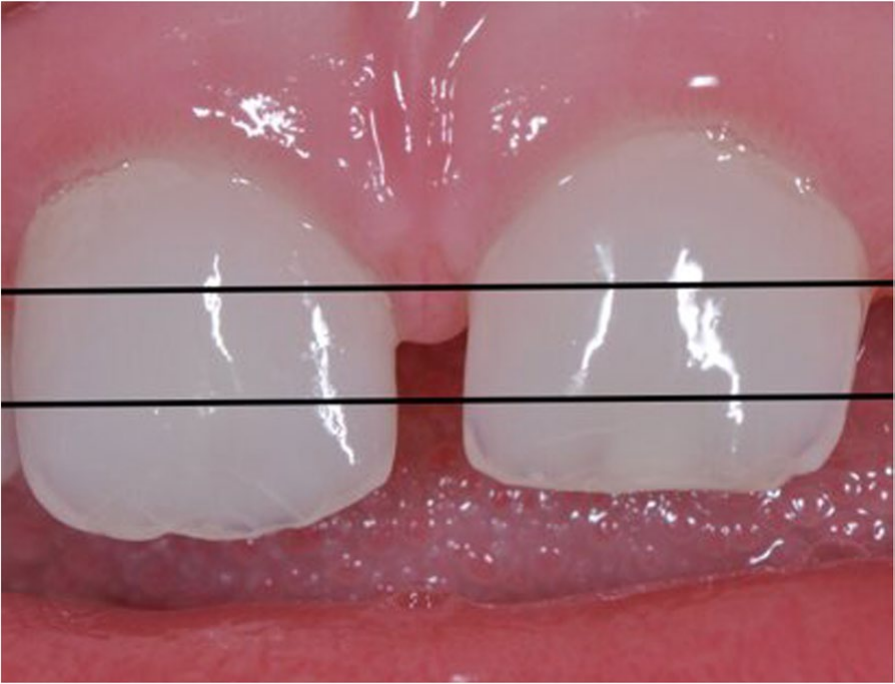
Nonoverlapping horizontal regions of PMCI teeth denoted by cervical (top region), middle (middle region), and incisal (bottom region)

The analysis of dental caries or tooth decay has been a major focus of recent work on modeling dental data [1–3]. While a dental caries focus is of major importance in dental research, the examination of enamel quantitative and qualitative defects that could also contribute at an early stage to caries formation is also of potential interest. In what follows we propose a set of methods that address different combinations of localized defects across different tooth regions of a child’s two front teeth, the primary maxillary central incisor teeth (PMCI), also referred to as teeth E and F. These PMCI teeth begin enamel calcification at approximately 14 gestational weeks [4]. PMCI teeth are fully calcified on average slightly over one-month postnatal [5, 6] with eruption into the oral cavity at approximately 1 year of age. Given this known duration of tooth development, these PMCI teeth provide an enamel record of exposures during pregnancy, at birth, and during postnatal that may impact the presence of localized developmental defects of the tooth enamel. The three major defects {enamel hypoplasia (EH), opacity (OP), and post-eruptive breakdown (PEB)}, which are illustrated in panels a, b, and c of Fig. 1, and dental caries (DC), treated as an enamel quantitative defect and shown in panel d of Fig. 1, were selected to compare and contrast enamel defect etiological predictors. Furthermore, the PMCI teeth are separated into three nonoverlapping horizontal regions (cervical, middle, and incisal) based on the general sequence of tooth development, which is shown in Fig. 2.

Our methods are motivated by our data to formulate a model that can handle examining how an individual defect is correlated across the regions of the PMCI teeth and examining the joint effect of the defects within a single region of the PMCI teeth. The motivating data were focused on assessing a model of mother and child factors present during pregnancy through delivery and early infancy indicative of the development of localized defects in the PMCI teeth. These data were obtained from maternal longitudinal data from a randomized, controlled trial (RCT) of maternal prenatal vitamin D_3_ supplementation [7], a follow-up study of the children [8], and the children's dental imaging data obtained at 2–5 years of age [9]. Maternal data were collected monthly from the 12th gestational week of pregnancy through delivery. Maternal predictors included: mother’s age; pre-pregnancy body mass index (BMI); number of months during pregnancy that antacids were taken; serum circulating concentrations at 12, 28, and 36 weeks of gestation of calcium (Ca), phosphorus (P), 25-hydroxyvitamin D (25OHD); and intact parathyroid hormone (PTH). The levels of OHD and PTH were used to create the functional vitamin D deficiency (FVDD) ratio (OHD / PTH) at 12, 28, and 36 weeks of gestation. These predictors have a natural correlation to one another; however, for the purposes of this study, we leave examination of their association at a single time point for future work.

The children's data were collected from birth through 4–6 weeks postnatal and at the time of their dental imaging visit once the child reached at least 2 years of age. Child predictors collected at birth through 4–6 weeks’ postnatal included: gestational age; early infancy diet (determined by whether the child had received formula by 4–6 weeks of age or was exclusively fed breastmilk); cord blood serum circulating Ca, P, 25OHD, PTH, and 1,25-dihydroxyvitamin D (1,25OH2D); and vitamin D binding protein (VDBP) genotype (focusing on 1s, 1f, and 2 genotypes). Cord blood 25OHD and PTH levels were used to create the FVDD at birth. Child predictors collected during the child’s dental imaging visit specific for dental caries include: the age of the child at the time of the imaging, whether the child had ever visited the dentist (DDS), whether fluoride varnish had been put on the child’s teeth (FLTX), child’s sex, and child’s salivary strep mutans count. The primary outcomes, EH, OP, PEB, and DC, were binary for the presence of a defect on the facial surfaces of the PMCI teeth in the cervical, middle, or incisal regions based on digital images from a digital camera (Nikon D90 SLR; Nikon Inc., Melville, NY) fitted with a ring flash and 105-mm macro lens with settings at f/32 aperture, 1/60 shutter speed, and a 3 × magnification.

Our analysis of the dental defect data is based on a Bayesian paradigm. Prior research in oral health that has utilized a Bayesian modeling approach varies depending on the research goal. Komarek et al. [10] implemented a modified version of the intensity model of Harkanen et al. [11] to examine how fluoride-intake affects the time to caries development for the permanent first molars. Bandyopadhyay et al. developed a random effect autologistic Bayesian regression model to assess the effects of exposures on a subject’s caries experience, [12] and multivariate spatial beta-binomial model count data that accounts for spatial associations in dental caries research [13]. Mutsvari et al. extended the multilevel autologistic Bayesian model to capture the probability of misclassification of caries presence on a surface of a tooth from a function of covariates, [14] and Jin et al. further extended the model to utilize a two-level Bayesian hierarchical model under spatial Markov random field assumptions to mitigate how the presence of dental caries at the tooth and surface level can be mixed complicating any analysis of the data under a unified framework. [15] In our modeling we assess the linkages between tooth region development and both the type of defects and the association with predictors that could be influential at different times during tooth development. In what follows we first develop our Bayesian methodology and propose different model formulations. After that we consider a simulation study that demonstrates the abilities of the approach in providing an appropriate modeling paradigm for the analysis of multiple defect occurrence on tooth regions. Finally, we provide a case study where we apply the methodology to defect and dental caries data scored from photographic images taken from a sample of children’s teeth [9].

## Methodology

We use Bayesian hierarchical models (BHMs) to assess the relation between defect presence and a range of relevant predictors [16]. This approach provides a flexible way to examine the different and complex relations between defects and tooth regions. We also employ special Bayesian variable selection methods (Gibbs variable selection: GVS) to examine all model combinations of predictors [17].

It is comparatively difficult to set up and fit non-Bayesian models when joint occurrences are to be examined or to allow for sophisticated model selection. In addition, BHMs allow the use of random effects so that extra noise in the outcome can be accommodated. In what follows we address the situation where we observe different compositions of defects within different tooth regions.

### Bayesian Model—$K^{th}$ Defect Across Regions

Assume we have $K$ defects and these are observed on surfaces of the child’s two front PMCI that are further divided into three regions: cervical, middle, and incisal. We assume that the observed presence of defect, $Y_{ijk}$, for the $i^{th}$ subject ($i = 1,...,n$) in the $j^{th}$ tooth region ($j = 1,2,3$), and $k^{th}$ defect follows a Bernoulli distribution as

$$Y_{ijk} \sim Bernoulli\left( {\pi_{ijk} } \right)$$

where $\pi_{ijk}$ is the probability of the $k^{th}$ defect in either of tooth E or F (notation of the Universal Numbering [18]) for the $i^{th}$ subject in the $j^{th}$ region. Furthermore, we denote ${\varvec{z}}_{ik}$ to represent the vector of logits or log-odds across the regions and assume it follows a multivariate normal distribution as

$$\varvec{z}_{ik}=\text{logit}\left[P\left(\varvec{Y}_{ik}=1\right)\right]\text{ = logit}(\varvec{\pi}_{ik})=\log\left(\frac{\varvec{\pi}_{ik}}{1-\varvec{\pi}_{ik}}\right)\sim MVN\left(\varvec{\mu}_{ik}, \varvec{\Sigma}_k\right)$$

where $\varvec{\mu}_{ik}$ represents the mean vector of the linear structure of predictors and ${\varvec{\varSigma}}_{k}$ is the $3 \times 3$ covariance matrix capturing the correlation between regions for a given defect. Notably, ${\varvec{\mu}}_{ik}$ is shown as

$$\mathop{{\varvec{\mu}}_{ik} }\limits_{(3 \times 1)} = {\varvec{\beta}}_{k}^{T} {\varvec{X}}_{i} + {\varvec{b}}_{ik}$$

where ${\varvec{X}}_{i} = \left( {1,x_{i1} ,x_{i2} ,...,x_{ip} } \right)^{T}$ represents a $(p + 1) \times 1$ vector of $p$ possible predictors, and ${\varvec{\beta}}_{k} = \left( {{\varvec{\beta}}_{0k} ,{\varvec{\beta}}_{1k} ,{\varvec{\beta}}_{2k} ,...,{\varvec{\beta}}_{pk} } \right)^{T}$ denotes a $(p + 1) \times 3$ matrix of fixed effects for each $j^{th}$ region. For each $k^{th}$ defect model, the $p^{th}$ fixed effect from the $j^{th}$ region is assumed to follow a normal distribution, $\beta_{pjk} \sim N(0,\sigma_{\beta }^{2} )$, with a hyperprior for $\sigma_{\beta }^{2}$ that follows a gamma distribution, $\sigma_{\beta }^{2} \sim Gamma(2,0.5)$. In addition, to capture the additional variation due to the regional clustering of the outcome variables, ${\varvec{b}}_{ik}$ is included as a linear term in the mean vector and is assumed to be independent with zero mean and follows a normal distribution prior, ${\varvec{b}}_{ik} \sim N(0,\sigma_{bk}^{2} )$, with a hyperprior for $\sigma_{bk}^{2}$ that follows a gamma distribution, $\sigma_{bk}^{2} \sim Gamma(2,0.5)$. This allows ${\varvec{\varSigma}}_{k}$ to account for the residual variability in the outcome variables not explained by the fixed and random effects.

### Bayesian Model – Multivariate Joint Model for $K$ Defects

Similar to the individual model across regions, we assume a joint model with $K$ defects observed on facial surface of the child subject’s two PMCI; however, this model is set within only one region. In our example, we examine a multivariate joint model where $j = 3$ for the incisal region. We assume the observed presence of defect follows a Bernoulli distribution, $Y_{ijk} \sim Bernoulli\left( {\pi_{ijk} } \right)$, where $\pi_{ijk}$ is the probability of the $k^{th}$ defect in either of tooth E or F for the $i^{th}$ subject in a set $j^{th}$ region. However, now we allow ${\varvec{z}}_{ij}$ to represent the vector of logits or log-odds of the joint defects and assume it follows a multivariate normal distribution as

$$\varvec{z}_{ij}=\text{logit}\left[P\left(\varvec{Y}_{ij}=1\right)\right]\text{ = logit}(\varvec{\pi}_{ij})=\log\left(\frac{\varvec{\pi}_{ij}}{1-\varvec{\pi}_{ij}}\right)\sim MVN\left(\varvec{\mu}_{ij},\varvec{\Sigma}_j\right)$$

where $\varvec{\mu}_{ij}$ represents the mean vector of the linear structure of predictors and ${\varvec{\varSigma}}_{j}$ is the $4 \times 4$ covariance matrix capturing the correlation between defects for a given region. Furthermore, $\varvec{\mu}_{ij}$ is shown as

$$\mathop {{\varvec{\mu}}_{ij} }\limits_{(4 \times 1)} = {\varvec{\beta}}_{j}^{T} {\varvec{X}}_{i} + {\varvec{b}}_{ij}$$

where ${\varvec{X}}_{i} = \left( {1,x_{i1} ,x_{i2} ,...,x_{ip} } \right)^{T}$ represents a $(p + 1) \times 1$ vector of $p$ possible predictors, and ${\varvec{\beta}}_{j} = \left( {{\varvec{\beta}}_{0j} ,{\varvec{\beta}}_{1j} ,{\varvec{\beta}}_{2j} ,...,{\varvec{\beta}}_{pj} } \right)^{T}$ denotes a $(p + 1) \times 4$ matrix of fixed effects for each $k^{th}$ defect. For each multivariate joint model in a $j^{th}$ region, the $p^{th}$ fixed effect for the $k^{th}$ defect follows a normal distribution, $\beta_{pjk} \sim N(0,\sigma_{\beta }^{2} )$, with a hyperprior for $\sigma_{\beta }^{2}$ that follows a gamma distribution, $\sigma_{\beta }^{2} \sim Gamma(2,0.5)$. To capture the added variation due to the clustering of the outcome variables within a subject, ${\varvec{b}}_{ij}$ was included as a linear term in the mean vector and assumed to be independent with zero mean following a normal distribution prior, ${\varvec{b}}_{ij} \sim N(0,\sigma_{bj}^{2} )$, with a hyperprior for $\sigma_{bj}^{2}$ that follows a gamma distribution, $\sigma_{bj}^{2} \sim Gamma(2,0.5)$. ${\varvec{\varSigma}}_{j}$ then accounts for the residual variability in the outcome variables not explained by the fixed and random effects.

### Covariance Structure

The size of the correlation structure depends on the whether we are implementing our model for an individual $k^{th}$ defect (which includes ${\varvec{\varSigma}}_{k}$ within the model) or a multivariate joint model in the $j^{th}$ region (which includes ${\varvec{\varSigma}}_{j}$). Using the Cholesky decomposition of the ${\varvec{\varSigma}}_{k}$ or ${\varvec{\varSigma}}_{j}$ prior into their respective scale and correlation matrix yields

$${\varvec{\varSigma}}_{k} = \left[ {\begin{array}{*{20}c} {\tau_{1} } & 0 & 0 & 0 \\ 0 & {\tau_{2} } & 0 & 0 \\ 0 & 0 & {\tau_{3} } & 0 \\ 0 & 0 & 0 & {\tau_{4} } \\ \end{array} } \right]{\varvec{\varOmega}}_{k} \left[ {\begin{array}{*{20}c} {\tau_{1} } & 0 & 0 & 0 \\ 0 & {\tau_{2} } & 0 & 0 \\ 0 & 0 & {\tau_{3} } & 0 \\ 0 & 0 & 0 & {\tau_{4} } \\ \end{array} } \right]{\text{ and }}{\varvec{\varSigma}}_{j} = \left[ {\begin{array}{*{20}c} {\tau_{1}^{*} } & 0 & 0 \\ 0 & {\tau_{2}^{*} } & 0 \\ 0 & 0 & {\tau_{3}^{*} } \\ \end{array} } \right]{\varvec{\varOmega}}_{j} \left[ {\begin{array}{*{20}c} {\tau_{1}^{*} } & 0 & 0 \\ 0 & {\tau_{2}^{*} } & 0 \\ 0 & 0 & {\tau_{3}^{*} } \\ \end{array} } \right]$$

where ${\varvec{\varOmega}}_{k}$ and ${\varvec{\varOmega}}_{j}$ are correlation matrices and $\tau_{k}$ as well as $\tau_{j}^{*}$ are coefficient scales, respectively. Both ${\varvec{\varOmega}}_{k}$ and ${\varvec{\varOmega}}_{j}$ each individually assume a LKJ prior distribution in their respective models where ${\varvec{\varOmega}}_{k} \sim LKJ\left( {\eta_{k} } \right)$ and ${\varvec{\varOmega}}_{j} \sim LKJ\left( {\eta_{j} } \right)$. $\eta_{k}$ and $\eta_{j}$ control how certain the prior is of large correlations between the regions within their respective models. If $\eta_{k} = 1$ or $\eta_{j} = 1$, then the density is uniform over the correlation matrix of a given order suggesting uncertainty of whether the regions are correlated. If $\eta_{k} > 1$ or $\eta_{j} > 1$, then extreme correlations are less likely, and if $0 < \eta_{k} < 1$ or $0 < \eta_{j} < 1$, then correlations between regions are favored though both positive and negative correlations are equally plausible [19].

The LKJ distribution is an extension of the D-vine method [20]. The D-vine method uniformly generates random correlation matrices over the space of all positive definite correlation matrices using an appropriate transformation of partial correlation that are then assigned to edges of a regular vine. The LKJ distribution, also referred to as the C-vine method, applies the assignments of partial correlations on the vine to the edges only on those partial correlations that have already been specified on the vine resulting in higher computational efficiency [21].

An alternative to the LKJ distribution could have been to allow ${\varvec{\varSigma}}_{k}$ or ${\varvec{\varSigma}}_{j}$to follow from the Wishart or inverse-Wishart distributions. These natural conjugate priors are common choices for a covariance matrix. However, these distributions are lacking flexibility to allow a wider range of uncertainty for variance parameters since they are constrained by a single degree of freedom [22]. In addition, since the marginal distribution for the variance of an inverse-Wishart is an inverse gamma distribution, datasets in which low variances are plausible can yield sensitive and biased inferences [23]. Lastly, there is a natural dependency between correlations and variances in the inverse-Wishart prior such that small variances are associated with correlations near zero and large variances are associated with correlations near one [24].

Additionally, within an individual defect models, another alternative could have been to allow ${\varvec{\varSigma}}_{k}$ to follow a spatial correlation structure in an individual defect model to examine the correlation between tooth regions. However, our goal was to implement a flexible model that could examine the correlation between regions, between defects, and in a nested model between both regions and defects. Thus, the correlation chosen for our model measures the pair-wise correlation between regions or between defects and not any spatial correlation within the PMCI teeth. Due to these reasons and the uncertainty of whether the regions are correlated, we assumed a LKJ prior for each $k^{th}$ defect such that ${\varvec{\varOmega}}_{k} \sim LKJ\left( 1 \right)$ in the individual defects models and a LKJ prior for each multivariate joint model in a $j^{th}$ region such that ${\varvec{\varOmega}}_{j} \sim LKJ\left( 1 \right)$. Furthermore, we assumed weakly informative priors on the scales, $\tau_{k} \sim Cauchy\left( {0,2.5} \right)$ and $\tau_{j}^{*} \sim Cauchy\left( {0,2.5} \right)$, which when combined with ${\varvec{\varOmega}}_{k}$ and ${\varvec{\varOmega}}_{j}$ formed both ${\varvec{\varSigma}}_{k}$ and ${\varvec{\varSigma}}_{j}$ respectively.

### Bayesian Variable Selection

To evaluate the possible number of alternative linear combinations of predictors, we employ a Bayesian variable selection method known as Gibbs variable selection. While other Bayesian variable selection approaches are available, they are beyond the scope of this paper. Let us examine how the variable selection method would work under an individual defects model for a $k^{th}$ defect across the regions. This would be similar for a multivariate joint model in a $j^{th}$ region except we would exchange subscripts accordingly.

We use an auxiliary indicator variable $I_{pj}$ (where $I_{pj} = 1$ indicates the presence of the $p^{th}$ predictor in the $j^{th}$ region while $I_{pj} = 0$ indicates the absence). Under this process, the mean vector of the linear structure of predictors is shown as

$$\mathop {{\varvec{\mu}}_{ik} }\limits_{(3 \times 1)} = {\varvec{\xi}}_{k}^{T} {\varvec{X}}_{i} + {\varvec{b}}_{ik}$$

where each element in the $(p + 1) \times 3$ matrix of ${\varvec{\xi}}_{k}$ is determined by ${\varvec{\xi}}_{pj} = \beta_{pj} I_{pj}$. Each indicator variable assumes a Bernoulli distribution, $I_{pj} \sim Bernoulli\left( {\psi_{pj} } \right)$, with a hyperprior for $\psi_{pj}$ that follows a Beta distribution, $\psi_{pj} \sim Beta\left( {\frac{1}{2},\frac{1}{2}} \right)$.

### Missingness (in Application)

Within our application, we assume the data to be missing at random (MAR) and address any missingness during model fitting. Since we fit our model for applications with non-missing analyses, our only missingness was encountered in our predictors. To handle this missingness, we assume that the predictors are a realization from a prior distribution. Any missing values in the predictors are then imputed as parameters and iteratively updated within our Bayesian computational approach.

For instance, let us examine how missingness would be addressed in an individual defect model across regions. We assumed continuous distributions to be normally distributed with a mean of the predictor’s non-missing values and the variance, $\sigma_{jk}^{2}$, to have a Gamma hyperprior where $\sigma_{jk}^{2} \sim Gamma\left( {2,0.5} \right)$. Binary predictors were assumed to follow a Bernoulli distribution with probability, $\nu_{jk}$, with a hyperprior for the probability to be $\nu_{jk} \sim Uniform\left( {0,1} \right)$. Last, for the one categorical predictor having three categories, VDBP, we imputed missing values using multinomial regression with maternal race as the predictor included in the model. The categories of maternal race included African American, Caucasian, and Hispanic. This decision was based on prior literature regarding the association between maternal race/ethnicity and genotype towards the type of the child’s VDBP.

$$VDBP_{i} \sim Multinomial(v_{1} ,v_{2} ,v_{3} )$$

$$v_{i} = \frac{{\zeta_{0} + Race_{i} \cdot \zeta_{1} }}{{\sum\limits_{i = 1}^{3} {\left( {\zeta_{0} + Race_{i} \cdot \zeta_{1} } \right)} }}$$

Each fixed effect in the linear function follows a normal distribution, $\zeta_{{p^{ * } j}} \sim N(0,\sigma_{\zeta }^{2} )$ with each having a hyperprior for $\sigma_{\zeta }^{2}$ that follows a Gamma distribution, $\sigma_{\zeta }^{2} \sim Gamma\left( {2,0.5} \right)$. The $p^{ * }$ denotes whether the fixed effect is the intercept, $p^{ * } = 0$, or the fixed effect for an individual’s maternal race, $p^{ * } = 1$.

### Model Computation

The simulation and application of our BHMs were both implemented with the R language [25] using MCMC simulations via the NIMBLE package [26]. This package is based on parsed versions of BUGS code; however, it extends the BUGS language for writing new functions or distributions yielding increased flexibility of model specification. Further, it compiles models using its own C +  + samplers increasing computational efficiency. All simulation and application models fit were run to convergence and confirmed using the Gelman-Rubin diagnostic.

## Case Studies - Application

We applied our methodology to dental defect data scored from photographic images made from a sample of children’s teeth to analyze the association of the presence of defects with potential predictors. We implemented our model for two scenarios: an individual defect model for the EH outcome to identify significantly associated predictors accounting for the correlation across the regions (cervical, middle, and incisal) of the child’s teeth; a multivariate joint model to identify predictors with a singular or joint association with defects (EH, OP, PEB, and DC) in the incisal region.

### Individual Enamel Hypoplasia Model

We fit a model across the three regions to examine how predictors are associated with EH. Though there were 161 total observations in the study sample, we only included observations with non-missing EH outcome measures. The sample size of 148 across the three regions for the model is shown in Table 1.

**Table 1:** Subjects per defect and region

	***Enamel Hypoplasia***
*Number of Subjects*	148
*Cervical* = *Yes (%)*	13 (8.97%)
*Middle* = *Yes (%)*	20 (13.89%)
*Incisal* = *Yes (%)*	43 (29.25%)

With the goal of distinguishing biomarkers associated with the presence of an EH defect in any of the cervical, middle, or incisal regions for the primary maxillary central incisors, we assumed to only have 1 defect where $k = 1$ for the EH defect. Thus, for the $i^{th}$ subject ($i = 1,...,n$) in the $j^{th}$ tooth region ($j = 1,2,3$), and $k = 1$ defect follows a Bernoulli distribution as

$$Y_{ij1} \sim Bernoulli\left( {\pi_{ij1} } \right)$$

where $\pi_{ij1}$ is the probability of the EH defect in either PMCI tooth for the $i^{th}$ subject in the $j^{th}$ region. ${\varvec{z}}_{i1}$ represents the vector of log-odds across the regions and is assumed to follow a multivariate normal distribution as

$$\varvec{z}_{i1}=\text{logit}\left[P\left(\varvec{Y}_{i1}=1\right)\right]\text{ = logit}(\varvec{\pi}_{i1})=\log\left(\frac{\varvec{\pi}_{i1}}{1-\varvec{\pi}_{i1}}\right)\sim MVN\left(\varvec{\mu}_{i1},\varvec{\Sigma}_1\right)$$

where $\varvec{\mu}_{i1}$ represents the mean vector of the linear structure of predictors and ${\varvec{\varSigma}}_{1}$ as the $3 \times 3$ covariance matrix capturing the correlation between regions for the EH defect.

We included all potential predictors except for those predictors specific to dental caries: DDS, FLTX, and child’s strep mutans count. We fit a full model to conduct Gibbs Variable Selection identifying those predictors with high posterior probabilities of inclusion in at least one region. This model indicated the child’s gestational age (incisal region), mother’s P at 36 weeks’ gestation (cervical region), mother’s FVDD ratio at 12 weeks’ gestation (cervical region), mother’s FVDD ratio at 28 weeks’ gestation (cervical region), and child’s age at dental imaging visit (cervical and incisal regions) were the only predictors to yield posterior probabilities of inclusion greater than 0.6 in those regions listed. We considered the use of the 0.5 threshold often proposed for Gibbs variable selection [27]. However, we needed to balance parsimony and explanatory power, and upon examination, we found the 0.6 threshold provided a better discriminatory performance. Using these predictors, we fit a final reduced model across all three regions.

Table 2 details the posterior parameter means on the odds scale and their 95% credible intervals for the reduced model fit across all three regions. Significant associations include the child’s gestational age (incisal region), mother’s FVDD ratio at 12 weeks (cervical region), and child’s age at visit (cervical and incisal regions). Interpretations for each predictor are similar pending on the predictor being examined. For gestational age, for example, the interpretation is as follows: Holding all other covariates constant, we expect to see an approximate 4% statistically non-significant higher odds of EH in the cervical region, an approximate 11% statistically non-significant lower odds of EH in the middle region, and an approximate 22% statistically significant lower odds of EH in the incisal region for a one week increase in gestational age.

**Table 2:** Enamel hypoplasia parameter estimates

	**Cervical Region**		**Middle Region**		**Incisal Region**	
	**Mean (SD)**	**95% CI**	**Mean (SD)**	**95% CI**	**Mean (SD)**	**95% CI**
**Intercept**	0.10 (2.09)	(0.03, 0.42)	0.24 (1.63)	(0.09, 0.61)	0.62 (1.42)	(0.30, 1.17)
**Gestational Age**	1.04 (1.18)	(0.77, 1.47)	0.89 (1.12)	(0.73, 1.11)	0.78 (1.11)	(0.63, 0.95)
**Mother’s P at 36 Weeks**	1.73 (1.74)	(0.66, 5.92)	1.35 (1.49)	(0.62, 3.12)	1.02 (1.41)	(0.52, 1.98)
**Mother’s FVDD at 12 Weeks**	0.33 (1.52)	(0.13, 0.68)	0.75 (1.24)	(0.48, 1.11)	0.82 (1.18)	(0.58, 1.10)
**Mother’s FVDD at 28 Weeks**	1.35 (1.17)	(1.01, 1.84)	0.87 (1.16)	(0.65, 1.17)	0.91 (1.12)	(0.73, 1.14)
**Child’s Age at Visit**	1.87 (1.39)	(1.06, 3.74)	1.28 (1.29)	(0.78, 2.15)	1.59 (1.28)	(1.02, 2.66)

**Fig. 3:**
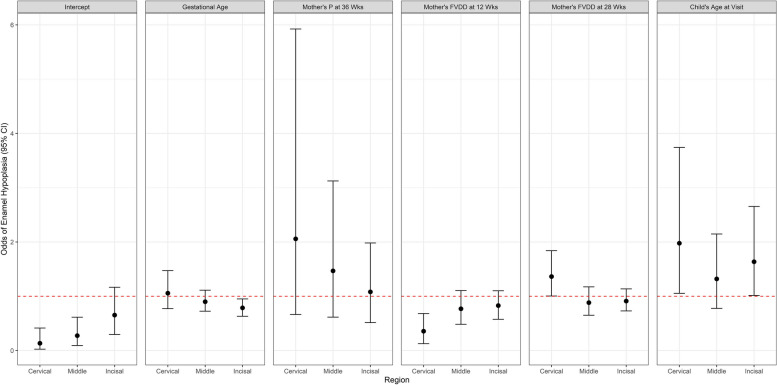
Enamel hypoplasia profile plot of predictors' 95% credible intervals on the odds scale for those predictors included in the reduced enamel hypoplasia model. The red dashed line denotes the null value of 1.0 on the odds scale

Furthermore, we observed a consistent directionality of the odds across each region for most predictors with the exceptions of mother’s FVDD ratio at 28 weeks’ gestation and child’s age at dental imaging visit, which have higher odds in the cervical and incisal regions relative to the middle region. Figure 3 depicts the directionality trend in odds across regions. There is a decreasing trend in odds across regions (cervical, middle, incisal) within predictors of the child’s gestational age and mother’s P at 36 weeks’ gestation. Conversely, there is an increasing trend in odds across regions within the predictor of mother’s FVDD ratio at 12 weeks. Additionally, while both predictors for mother’s FVDD ratio at 12 weeks and child’s age at imaging were only statistically significant in the cervical region, the bounds of their respective credible intervals were nearly significant.

Moreover, we examined the posterior correlation between regions shown in Table 3. The diagonal of the correlation matrix between effects degrades due to the LKJ prior distribution where each column indicates the degradation occurring; however, a simple interpretation can be made in the first column as measures indicate a one-to-one correlation between regions. These results reflect a non-statistically significant correlation between the cervical region and either the middle or incisal regions, and although the degradation is present, one can intuitively notice that the correlation between the middle and incisal regions is also not statistically significant.

**Table 3:** Correlation between regions (Enamel Hypoplasia)

	**Cervical**	**Middle**	**Incisal**
Cervical	1.00 (1.00, 1.00)	0.00 (0.00, 0.00)	0.00 (0.00, 0.00)
Middle	0.20 (-0.77, 0.89)	0.85 (0.43, 1.00)	0.00 (0.00, 0.00)
Incisal	0.04 (-0.88, 0.89)	0.01 (-0.85, 0.84)	0.71, (0.21, 0.99)

### Multivariate Defects Model

We fit a joint model at the incisal region to examine how predictors are associated under a joint effect for all defects (EH, OP, PEB, and DC). Any observation with the presence of at least one known defect in their PMCI regardless of whether that information was missing for any other defect was included in the model, as summarized in Table 4 below.

**Table 4:** Subjects per defect in incisal region

	***Incisal Region***
*Number of Subjects*	159
*Enamel Hypoplasia* = *Yes (%)*	43 (29.25%)
*Opacity* = *Yes (%)*	20 (13.89%)
*Post-Eruptive Breakdown* = *Yes (%)*	70 (47.95%)
*Dental Caries* = *Yes (%)*	23 (15.23%)

We assumed a joint model with 4 defects where $k = 1,2,3,4$ that represent the defects as 1 (EH), 2 (OP), 3 (PEB), and 4 (DC). Thus, for the $i^{th}$ subject ($i = 1,...,n$) in the $j = 3$ incisal region with the $k^{th}$ defect, our model follows a Bernoulli distribution as

$$Y_{i3k} \sim Bernoulli\left( {\pi_{i3k} } \right)$$

where $\pi_{i3k}$ is the probability of the joint presence of a defect in any PMCI for the $i^{th}$ subject in the incisal region. ${\varvec{z}}_{i3}$ represents the vector of logits of the joint defects and we assumed it follows a multivariate normal distribution as

$$\varvec{z}_{i3}=\text{logit}\left[P\left(\varvec{Y}_{i3}=1\right)\right]\text{ = logit}(\varvec{\pi}_{i3})=\log\left(\frac{\varvec{\pi}_{i3}}{1-\varvec{\pi}_{i3}}\right)\sim MVN\left(\varvec{\mu}_{i3},\varvec{\Sigma}_3\right)$$

where $\varvec{\mu}_{i3}$ represents the mean vector of the linear structure of predictors in the incisal region and ${\varvec{\varSigma}}_{3}$ as the $3 \times 3$ covariance matrix capturing the correlation between defects in the incisal region.

With dental caries as an outcome in the joint model, the potential predictors from the imaging visit were also included. We fit a full model to conduct Gibbs Variable Selection identifying those predictors with high posterior probabilities of inclusion for at least one defect. This model indicated that the child’s gestational age (EH and OP), whether the child was ever on formula (OP and DC), mother’s BMI (PEB), mother’s Ca at 28 weeks’ gestation (EH), mother’s P at 28 weeks’ gestation (PEB), mother’s FVDD ratio at 36 weeks’ gestation (OP), child’s age at dental imaging visit (EH and PEB), and child’s sex (OP) were the only predictors to yield posterior probabilities of inclusion greater than 0.6 in those regions listed. Similar to the individual model, we found that the 0.6 threshold yielded an improved discriminatory performance relative to the 0.5 threshold typically used for Gibbs variable selection [27]. Using these predictors, we fit a final reduced joint model for all defects within the incisal region.

**Fig. 4:**
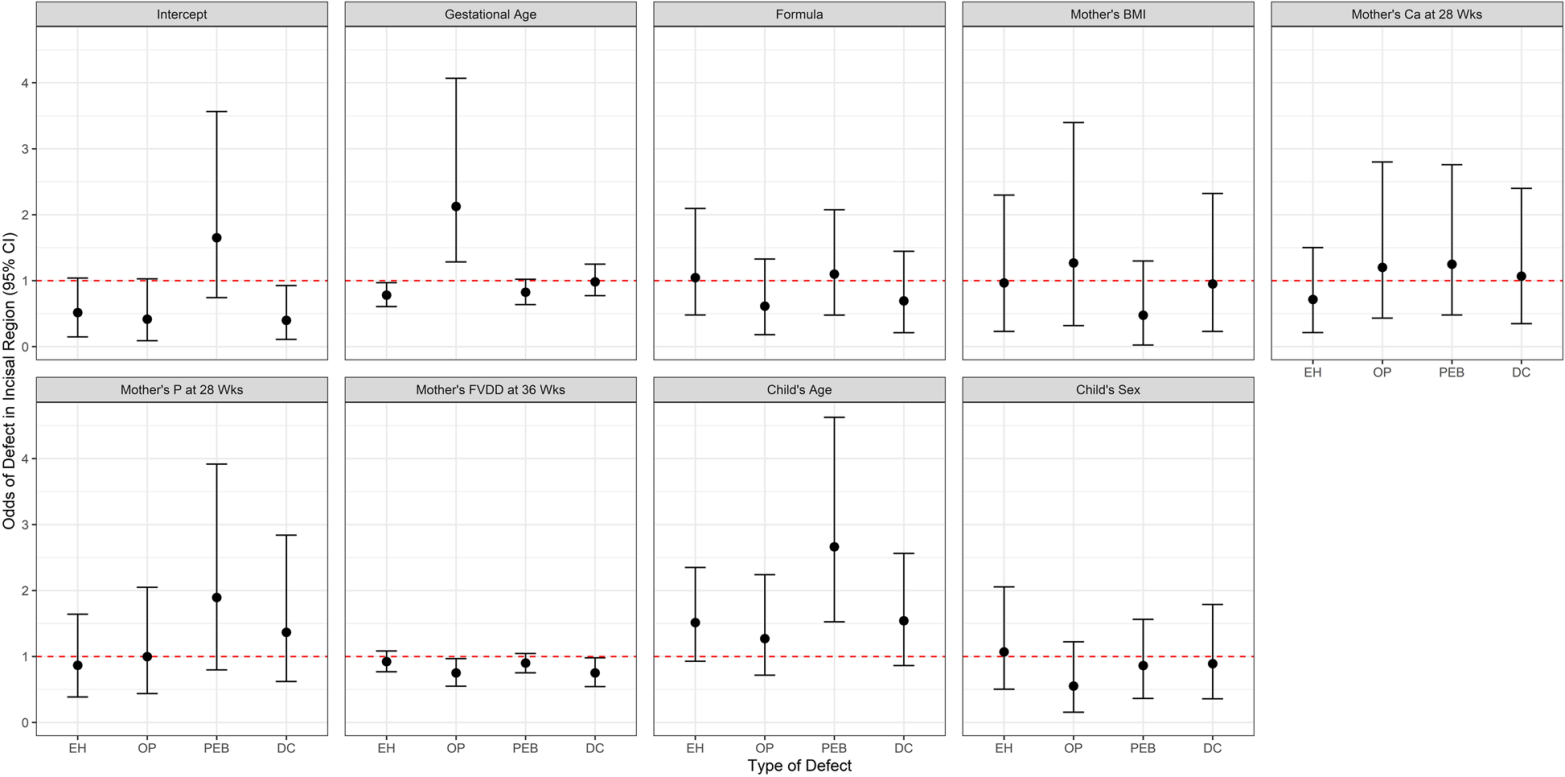
Incisal region profile plot of predictors' 95% credible intervals on the odds scale for those predictors included in the reduced joint model of all defects in the incisal region. The red dashed line denotes the null value of 1.0 on the odds scale

Table 5 details the posterior parameter means on the odds scale and their 95% credible intervals for the reduced joint model of all defects. Significant associations include the child’s gestational age (EH and OP), mother’s FVDD ratio at 36 weeks’ gestation (OP and DC) and child’s age at dental imaging visit (PEB). Each predictor’s interpretation is similar, and taking the child’s gestational age as an example, the interpretation is as follows: Holding all other covariates constant, we expect to see an approximate 22% statistically significant lower odds of EH, an approximate two-fold statistically significant higher odds of OP, an approximate 18% statistically non-significant lower odds of PEB, and an approximate 3% statistically non-significant lower odds of DC in the incisal region for a one week increase in gestational age. The joint relationship between posterior parameter means across the defects is shown in Fig. 4 below. Of note, child’s gestational age displays a statistically significant higher odds of OP holding all other predictors constant whereas its association with the remaining outcomes has statistically non-significant lower odds. Conversely, child’s age at time of the dental imaging visit indicates higher odds of any defect when holding all other predictors constant, although it is only a statistically significant higher odds of PEB.

**Table 5:** Parameter estimates (all defects joint model)

	***Enamel Hypoplasia***		***Opacity***		***Post-Erupt. Break***		***Dental Caries***	
	Mean (SD)	95% CI	Mean (SD)	95% CI	Mean (SD)	95% CI	Mean (SD)	95% CI
***Intercept***	0.46 (1.62)	(0.15, 1.04)	0.35 (1.87)	(0.09, 1.03)	1.51 (1.50)	(0.74, 3.56)	0.35 (1.74)	(0.11, 0.93)
***Gestational Age***	0.78 (1.13)	(0.61, 0.97)	2.03 (1.33)	(1.28, 4.07)	0.82 (1.13)	(0.64, 1.02)	0.97 (1.13)	(0.77, 1.25)
***Formula***	0.97 (1.46)	(0.48, 2.10)	0.54 (1.67)	(0.18, 1.33)	1.02 (1.46)	(0.48, 2.08)	0.62 (1.64)	(0.21, 1.45)
***Mother’s BMI***	0.82 (1.78)	(0.23, 2.30)	1.04 (1.81)	(0.32, 3.40)	0.33 (2.77)	(0.02, 1.30)	0.82 (1.77)	(0.23, 2.32)
***Mother’s Ca (28 Wks)***	0.64 (1.64)	(0.21, 1.50)	1.07 (1.60)	(0.43, 2.80)	1.13 (1.55)	(0.48, 2.76)	0.96 (1.61)	(0.35, 2.40)
***Mother’s P (28 Wks)***	0.81 (1.43)	(0.39, 1.64)	0.92 (1.48)	(0.44, 2.05)	1.74 (1.50)	(0.80, 3.92)	1.27 (1.47)	(0.62, 2.84)
***Mother’s FVDD (36 Wks)***	0.92 (1.09)	(0.77, 1.08)	0.74 (1.16)	(0.55, 0.97)	0.90 (1.09)	(0.75, 1.05)	0.74 (1.16)	(0.54, 0.98)
***Child’s Age at Visit***	1.47 (1.27)	(0.93, 2.35)	1.22 (1.34)	(0.72, 2.24)	2.56 (1.32)	(1.53, 4.63)	1.48 (1.32)	(0.86, 2.56)
***Child’s Sex***	1.00 (1.44)	(0.51, 2.06)	0.48 (1.70)	(0.16, 1.22)	0.81 (1.43)	(0.36, 1.56)	0.82 (1.50)	(0.36, 1.79)

Furthermore, we examined the posterior correlation between defects shown in Table 6. The diagonal of the correlation matrix between effects degrades due to the LKJ prior distribution where each column indicates the degradation occurring; however, a simple interpretation can be made in the first column as measures indicate a one-to-one correlation between defects. These means indicate a near statistically significant positive correlation between EH and PEB.

**Table 6:** Correlation between defects (incisal region)

	**EH**	**OP**	**PEB**	**DC**
EH	1.00 (1.00, 1.00)	0.00 (0.00, 0.00)	0.00 (0.00, 0.00)	0.00 (0.00, 0.00)
OP	-0.35 (-0.87, 0.50)	0.85 (0.48, 1.00)	0.00 (0.00, 0.00)	0.00 (0.00, 0.00)
PEB	0.56 (-0.06, 0.91)	-0.25 (-0.85, 0.44)	0.63 (0.22, 0.97)	0.00 (0.00, 0.00)
DC	0.49 (-0.22, 0.92)	-0.05 (-0.76, 0.67)	0.15 (-0.57, 0.82)	0.55 (0.18, 0.93)

## Analysis - Simulation Study

A simulation study was implemented to evaluate the ability of our model to consistently identify predictors associated with the presence of a defect in any region and how accurate the obtained posterior predictor estimates relate to the predictor estimates used in the data generation. Random datasets were generated for each iteration using estimates obtained from the original application model. The summary statistics for each defect in the application model and the assumed distributions for each predictor are shown in Table S1 in the supplementary section.

Due to the complexity of the BHM, we constructed three different simulation scenarios based on the individual defect model for EH, OP, PEB, and DC. The first simulation scenario re-fit the original application model for a simulated set of predictors and outcomes given the posterior means of the parameters and the covariance structure from the original application model. The parameter estimates on the log-odds scale (shown in Table 7 for EH) and the covariance structure (shown in Table 8 for EH) are under the Scenario 1 section. The purpose of this scenario was to examine the consistency of our model identifying predictors with significant inclusion and the predictors’ posterior means. The second simulation scenario assumed that the regions were independent of one another (under Scenario 3 in Table 7 for EH) with the purpose of whether our model could identify a different covariance structure relative to the original application. The third simulation scenario assumed only a select number of predictors had a log-odds magnitude greater than 0. The number of non-zero parameter estimates was based on the number of predictors included in the reduced models for each individual defect in the original application. The magnitudes shown in Tables 7 and 8 are based on the individual defect model for enamel hypoplasia, and the other defect models are shown in Tables S2-S6 in the Supplementary section.

**Table 7:** Parameter magnitudes set for each simulation scenario (EH)

	***Scenario 1***			***Scenario 2***			***Scenario 3***		
	Cervical	Middle	Incisal	Cervical	Middle	Incisal	Cervical	Middle	Incisal
*Intercept*	-1.235	-2.132	-0.383	-1.235	-2.132	-0.383	0.5	0.25	0.75
*Child’s OH2D*	0.001	0.007	0.014	0.001	0.007	0.014	0	0	0
*Child’s Ca*	0.129	0.029	-0.067	0.129	0.029	-0.067	0.4	0.8	0.6
*Child’s P*	-0.167	-0.071	-0.171	-0.167	-0.071	-0.171	0	0	0
*Gestational Age*	0.026	-0.04	-0.198	0.026	-0.04	-0.198	0	0	0
*Formula*	-0.443	-0.101	-0.007	-0.443	-0.101	-0.007	0	0	0
*Mother’s Age*	0.036	0.009	-0.009	0.036	0.009	-0.009	1.8	0.6	1.4
*Mother’s BMI*	0.055	-0.291	-0.174	0.055	-0.291	-0.174	0	0	0
*Antacid Counts*	-0.058	-0.097	-0.121	-0.058	-0.097	-0.121	0	0	0
*Mother’s Ca at 12 Wks*	-0.046	0.052	0.064	-0.046	0.052	0.064	0	0	0
*Mother’s Ca at 28 Wks*	-0.013	-0.067	-0.312	-0.013	-0.067	-0.312	0	0	0
*Mother’s Ca at 36 Wks*	0.084	0.11	-0.06	0.084	0.11	-0.06	0	0	0
*Mother’s P at 12 Wks*	0.064	-0.293	-0.003	0.064	-0.293	-0.003	0	0	0
*Mother’s P at 28 Wks*	0.052	0.028	-0.059	0.052	0.028	-0.059	0.6	0.4	0.8
*Mother’s P at 36 Wks*	0.398	0.081	0.007	0.398	0.081	0.007	0	0	0
*Child’s FVDD*	0.014	-0.001	0.009	0.014	-0.001	0.009	0	0	0
*Mother’s FVDD at 12 Wks*	-1.003	-0.105	-0.092	-1.003	-0.105	-0.092	0.45	0.75	0.65
*Mother’s FVDD at 28 Wks*	0.118	-0.011	-0.046	0.118	-0.011	-0.046	0	0	0
*Mother’s FVDD at 36 Wks*	-0.043	-0.015	-0.047	-0.043	-0.015	-0.047	0	0	0
*Vit-D (Cat. 1)*	-0.061	-0.173	-0.106	-0.061	-0.173	-0.106	1.1	0.9	1.3
*Vit-D (Cat. 2)*	-0.023	0.058	-0.073	-0.023	0.058	-0.073	0	0	0
*Child’s Age at Visit*	0.628	0.12	0.297	0.628	0.12	0.297	0	0	0
*Child’s DDS*	-0.332	-0.278	-0.121	-0.332	-0.278	-0.121	0	0	0
*Child’s Fltx*	-0.001	0.173	-0.101	-0.001	0.173	-0.101	0	0	0
*Child’s Sex*	0.01	-0.039	0.003	0.01	-0.039	0.003	0	0	0
*Child’s Strep Mutans Cnt*	0.036	0.692	-0.003	0.036	0.692	-0.003	1	2	1.5

Description: Parameter magnitudes used for Scenarios 1 and 2 are based on the posterior means of the parameters from original application model for the enamel hypoplasia defect. Parameter magnitudes for Scenario 3 are randomly assigned with the number of predictors chosen based on the number of predictors that met the threshold for inclusion in the original application model for enamel hypoplasia

**Table 8:** Magnitudes used for covariance structure (EH)

	***Scenario 1***			***Scenario 2***			***Scenario 3***		
	Cervical	Middle	Incisal	Cervical	Middle	Incisal	Cervical	Middle	Incisal
*Cervical*	1.0000	0.1685	0.0715	1.0000	0.0000	0.0000	1.0000	0.1685	0.0715
*Middle*	0.1685	0.8601	0.0200	0.0000	1.0000	0.0000	0.1685	0.8601	0.0200
*Incisal*	0.0715	0.0200	0.7311	0.0000	0.0000	1.0000	0.0715	0.0200	0.7311

Description: Estimates for the covariance matrix obtained for Scenarios 1 and 3 are based on the posterior means of the LKJ covariance structure from original application model for the enamel hypoplasia defect. Estimates for the covariance matrix for Scenario 2 are under the assumption that the regions are independent

We fit 50 iterations for each individual model over the three scenarios collecting 2,000 samples after a burn-in of 400,000. We display the results for the simulations for the EH individual defect model; however, we provide additional results for the other defects in the Supplementary section.

### Scenario 1: Re-fitting Original Application

**Fig. 5:**
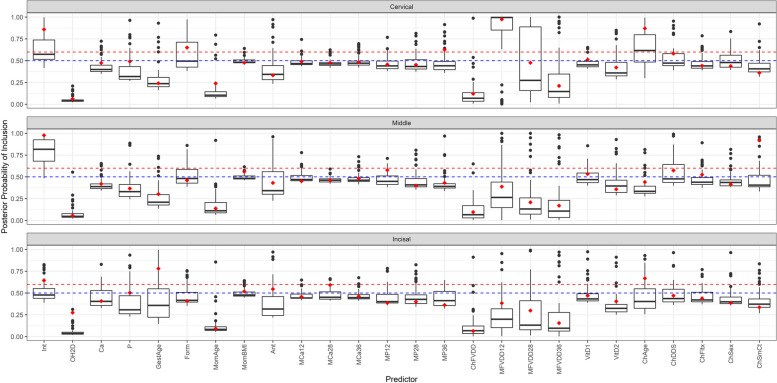
Displays the median and range of the posterior probabilities of inclusion across the 50 iterations of the simulation for enamel hypoplasia. The horizontal red dashed line denotes the stricter threshold of inclusion (set at 0.6) based on the large number of predictors in our model. The horizontal blue dashed line denotes the original threshold of inclusion at 0.5. The red points shown in each box plot are the full models’ posterior probability of inclusion from the original application for that predictor in that region

Under this scenario, we compared the range of the posterior probabilities of inclusion for each of the predictors obtained from the simulation with their posterior probability of inclusion from the original application model, which is shown below in Fig. 5 (EH) and Figures S3 (OP), S7 (PEB) and S11 (DC) found in the Supplementary section. We observed that nearly all predictors that had high posterior probabilities of inclusion greater than 0.85 in the original application model had inclusion ranges that either included or were exclusively greater than the 0.6 threshold. One exception was in models where the child strep mutans count predictor was originally identified as inclusive in the original application model; however, the range of posterior probabilities of inclusion had difficulty identifying this predictor as inclusive with ranges less than the 0.6 threshold. This difference between the simulations and application was likely caused by the original application’s dataset having an upper limit of three strep mutans count; however, our generated data did not truncate at that upper limit, resulting in more variability between the original and simulated data. Our results also showed that predictors that had posterior probabilities of inclusion between 0.6 and 0.85 in the original application model had ranges that varied across both the 0.5 and 0.6 thresholds. More notably, however, non-inclusive predictors from the original application model had posterior probability of inclusion ranges less than 0.6 indicating consistency in not picking up non-inclusive predictors for our reduced models.

Given a predictor returning a posterior probability of inclusion greater than the 0.6 threshold at any iteration of the simulation, the predictor would be used to fit a reduced model while non-inclusive predictors would be removed. Under this premise, we obtained the posterior parameter means on the odds scale for each predictor in a reduced model setting given that they were an inclusive predictor at that iteration. We observed that inclusive predictors had posterior parameter mean ranges had a more noticeable separation from the null odds the higher the posterior probability of inclusion. Furthermore, non-inclusive predictors had posterior parameter mean ranges closely centered around the null odds. These are shown in the Supplementary section in Figures S1 (EH), S4 (OP), S8 (PEB), and S12 (DC).

**Fig. 6:**
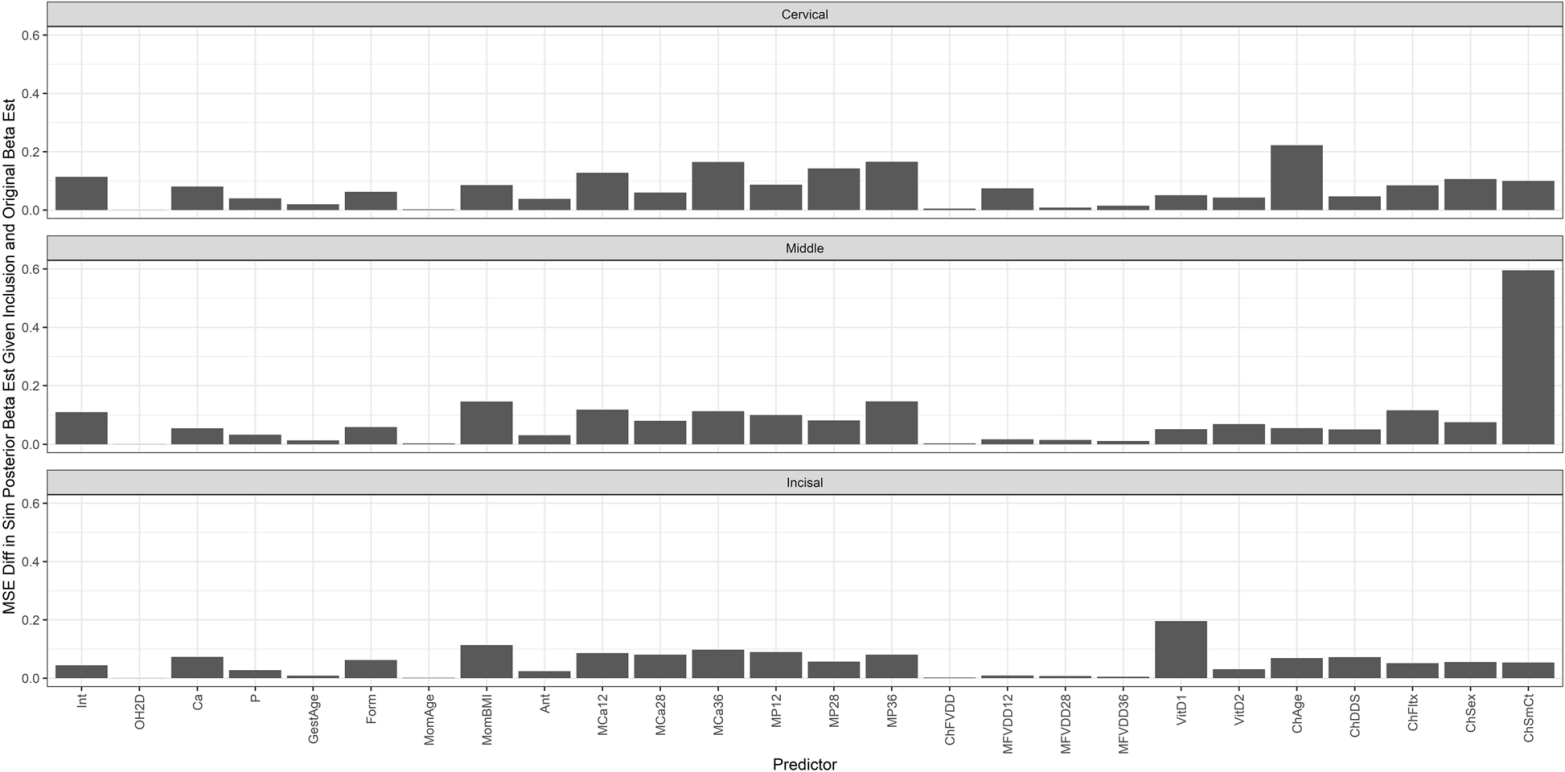
MSE in posterior parameter estimates given inclusion (EH). Description: Depicts the mean-squared error between the original application’s posterior parameter means and the posterior means obtained from the iterations of the simulation given their inclusion for enamel hypoplasia

Finally, we analyzed the error between the posterior parameter means at each iteration (given that they were included in the original application model) and their posterior parameter means from the original application model. The models performed well with small errors for nearly all predictors. The exceptions occurred when the child’s strep mutans count predictor was an inclusive predictor in the original application model, which we noted prior as to why that was likely the case. We display the mean-squared error (MSE) results for EH in Fig. 6 below to evaluate the goodness. Additional results for MSE and mean absolute error (MAE) are shown in the Supplementary section in Figure S2 (EH MAE), Figure S5 (OP MSE), Figure S6 (OP MAE), Figure S9 (PEB MSE), Figure S10 (PEB MAE), Figure S13 (DC MSE), and Figure S14 (DC MAE). The choice to use MSE and MAE over DIC and WAIC was made to examine the differences between the predicted posterior parameter means from the simulations, and we leave the alternative measures for model selection.

### Scenario 2: Independent Regions

Since there were not enough outcomes of interest in two regions for the original application of the individual PEB model, we ran this simulation scenario for only three individual defect models (EH, OP, and DC).

While our goal was to examine the performance of our model to pick up the covariance structure under a different structure relative to the original application model, we also obtained results for how well the models identified posterior probabilities of inclusion and posterior parameter means. Our inferences were similar to what we returned in Scenario 1 for each predictor. The results for the range of posterior probabilities of inclusion are shown in Figures S15 (EH), S18 (OP) and S22 (DC) in the Supplementary section. The results for the posterior parameter means given that the predictors had posterior probabilities of inclusion greater than 0.6 are shown in Figures S16 (EH), S19 (OP) and S23 (DC) in the Supplementary section.

**Fig. 7:**
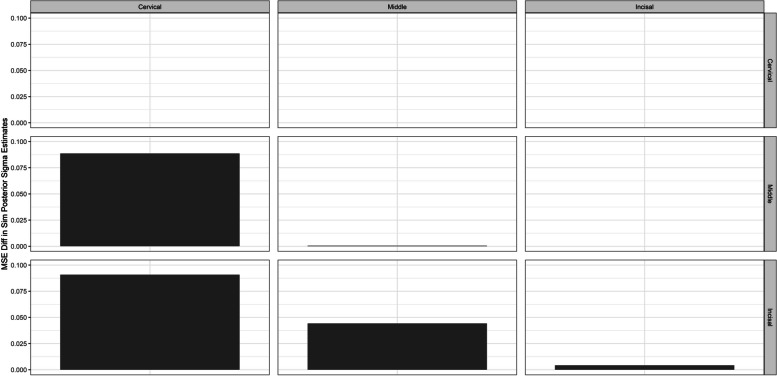
Details the mean-squared error between the original application’s posterior means of the covariance structure for the LKJ correlation and the posterior means of the covariance structure obtained from the iterations of the simulation for enamel hypoplasia

The main goal of this scenario, however, was to examine how well our model adjusted to a different correlation structure. Under the LKJ covariance structure, we obtained the error between our assumption of independent regions and the posterior means of covariance. For the EH model, we can note that there are small differences in the assumed covariance structure and the posterior means we returned from our model. The MSE difference between the two structures was small as evidenced by the results shown below in Fig. 7 (EH MSE). Additional figures are shown in the Supplementary section under Figure S17 (EH MAE), Figure S20 (OP MSE), Figure S21 (OP MAE), Figure S24 (DC MSE), and Figure S25 (DC MAE).

### Scenario 3: Adjusting Posterior Means

**Fig. 8:**
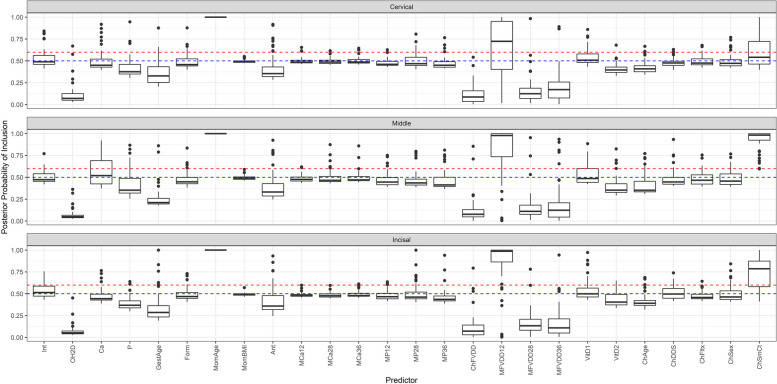
Depicts the median and range of the posterior probabilities of inclusion across the 50 iterations of the simulation for enamel hypoplasia. The horizontal red dashed line denotes the stricter threshold of inclusion (set at 0.6) based on the large number of predictors in our model. The horizontal blue dashed line denotes the original threshold of inclusion at 0.5

Under this scenario, our main goal was to examine how well our model was able to identify predictors that we generated to have non-zero magnitudes of varying strength as inclusive as well as determine their appropriate magnitudes. The range of the posterior probabilities of inclusion for each of the predictors obtained from the simulation was compared with the set posterior means from Table 7, and these results are shown below in Fig. 8 (EH) as well as Figures S29 (OP), S33 (PEB), and S37 (DC) in the Supplementary section. In general, non-zero magnitude predictors had more variable posterior probabilities of inclusion ranges that were also higher than null predictors. Notably, the null predictors’ posterior probability of inclusion ranges were at or below the 0.5 threshold, indicating that the model did not inflate the association between null predictors and the defect. One exception to these results was the mother’s P at 28 weeks, which consistently had lower posterior probabilities of inclusion. This could be due to the natural correlation it shares with the other maternal P predictors at 12 and 36 weeks resulting in its true inclusion being diminished.

Conditioning on a predictor being inclusive in the iteration of the simulation, we obtained the posterior parameter means on the odds scale for each predictor in a reduced model. Inclusive predictors had posterior parameter mean ranges that had distinct separation from the null odds with evidence that the more inclusive a predictor is in the model, the greater the separation from the null odds. Furthermore, non-inclusive predictors had posterior parameter mean ranges closely centered around the null odds. These are shown in the Supplementary section in Figures S26 (EH), S30 (OP), S34 (PEB), and S38 (DC).

Unlike the other scenarios, we observed more inclusive predictors having greater error between the set posterior parameter means and the posterior parameter means obtained from the simulations. While the child’s strep mutans count predictor was problematic throughout all simulations, the mother’s age predictor had an increased amount of error between the posterior parameter means relative to the other scenarios. Ultimately, the models performed well with small errors for the other predictors. We display these results in the Supplementary section in Figure S27 (EH MSE), Figure S28 (EH MAE), Figure S31 (OP MSE), Figure S32 (OP MAE), Figure S35 (PEB MSE), Figure S36 (PEB MAE), Figure S39 (DC MSE), and Figure S40 (DC MAE).

## Discussion

The underlying theme of the Bayesian model we proposed is to develop a model for spatially multivariate dental defects and dental caries assuming a covariance structure that handles spatial and joint effects. Our goal for this theme is that the model proposed is consistent with alternative models that assume different covariance structures, which avoids needing to use separate models under more rigid covariance structures to model the same datasets under different scopes. By consistent, we mean that inferences made from models remain steady in identifying the appropriate posterior probabilities of inclusion and posterior covariance means under differing scenarios.

Our approach is different from other multivariate or spatial models, which use alternative covariance structures to identify the correlation between joint or spatial effects. Those assumed covariance structures are powerful and commonly used, but they are limited in their rigidness to account for wider uncertainty for variance parameters. An advantage of our approach is to implement a correlation structure with increased flexibility to allow for wider uncertainty for variance parameters while including a tuning parameter that provides greater information to control how certain our prior is of large correlations between regions or joint effects. Ideally, our approach will be extended further to investigate the correlation between both regions and joint effects within the same model.

In the simulation scenarios explored in this paper, our model was consistent in identifying the posterior probabilities of inclusion relative to the results from the original application model and when adjusting the posterior parameter means. Predictors with posterior probabilities of inclusion greater than 0.85 in the original application model had a range of iterations above the 0.6 strict threshold for inclusion under Gibbs variable selection. Furthermore, predictors with posterior probabilities of inclusion between 0.6 and 0.85 in the original application model had posterior inclusion ranges that spanned the 0.5 and 0.6 thresholds. One exception was the child strep mutans count predictor, which could be explained by not truncating the upper limit of the predictor when generating data under the simulation scenario. Additionally, our model adjusted to independent correlations between regions under similar iterations of our generated data.

## Conclusion

We proposed and evaluated a set of Bayesian hierarchical models to address the appearance of different combinations of defects across different tooth regions. In our modeling we assess the linkages between tooth region development and both the type of defect and associations with etiological predictors of the defects which could be influential at different times during the tooth crown development. We use BHMs to assess the relation between defect presence and a range of relevant predictors and employ GVS to examine all model combinations of predictors to select a subset of relevant predictors. We assumed a LKJ prior for each model to allow for a wider range of uncertainty in variance parameters and to yield a natural extension to a nested hierarchical model.

The developments in this paper shed new light on methods for capturing inclusive predictors in multivariate joint models or spatial models under the same covariance structure. Both the models fit for an individual developmental enamel defect accounting of the correlation between regions and the joint model for the multivariate developmental enamel defects within a single region yielded inference on subsets of etiological biomarkers associated with the respective outcomes in respective regions. Moreover, these models indicated the correlation between defects and regions in their respective models. The proposed model provides a natural extension to expanding the covariance structure to account for a region by joint defects covariance structure.

### Supplementary Information

**Supplementary Material 1.** 

## Data Availability

The method is implemented using the nimble R package at https://cran.r-project.org/web/packages/nimble/index.html. The analysis and simulation scripts are available at https://github.com/everparkeller/Bayesian_Modeling_Spatial_Defects. The datasets used and/or analysed during the current study are available from the corresponding author on reasonable request.

## Abbreviations

PMCI: Primary maxillary central incisor
EH: Enamel hypoplasia
OP: Opacity
PEB: Post-eruptive breakdown
DC: Dental caries
RCT: Randomized, controlled trial
Ca: Calcium
P: Phosphorus
OHD: 25-Hydroxyvitamin D
PTH: Intact parathyroid hormone
OH2D: 1,25-Dihydroxyvitamin D
VDBP: Vitamin-D binding protein
FVDD: Functional vitamin-D deficiency
DDS: Whether child had ever visited dentist
FLTX: Whether fluoride varnish had been put on child’s teeth
BHM: Bayesian hierarchical model
GVS: Gibbs variable selection
MCMC: Markov Chain Monte Carlo
LKJ: Lewandowski-Kurowicka-Joe
