# Supplementary material for: Bayesian modeling of spatially differentiated multivariate enamel defects of the children’s primary maxillary central incisor teeth
Source: BMC Med Res Methodol. 2024 Apr 15;24:88. doi: 10.1186/s12874-024-02211-8 (PMC11017560; doi:10.1186/s12874-024-02211-8)
Supplement: Supplementary file 1 — Supplementary Material 1. [file 12874_2024_2211_MOESM1_ESM.docx]

**Supplementary**

***Table S 1 - Summary Statistics of Predictors from Original Model and Simulated Distributions***

|  | **Observed Summary Statistics from Original Data** | | | | **Sim.  Dist.** |
| --- | --- | --- | --- | --- | --- |
|  | **EH** | **OP** | **PEB** | **DC** |  |
| **Number of Subjects** | 148 | 147 | 146 | 152 | 161 |
| **Maternal** |  |  |  |  |  |
| Age [Mean (SD)] | 27.63 (5.53) | 27.65 (5.55) | 27.67 (5.55) | 27.47 (5.62) | $N\left(\mu_{k},\sigma_{k}^{2}\right)$ |
| BMI [Mean (SD)] | 3.33 (0.23) | 3.33 (0.22) | 3.33 (0.23) | 3.32 (0.23) | $N\left(\mu_{k},\sigma_{k}^{2}\right)$ |
| Ant. [Count (%)] |  |  |  |  | $Poisson\left(\lambda_{k}\right)$ |
| 0 | 72 (48.65) | 71 (48.3) | 71 (48.63) | 74 (48.68) |  |
| 1 | 20 (13.51) | 20 (13.61) | 20 (13.7) | 19 (12.5) |  |
| 2 | 20 (13.51) | 19 (12.93) | 19 (13.01) | 22 (14.47) |  |
| 3 | 10 (6.76) | 11 (7.48) | 10 (6.85) | 11 (7.24) |  |
| 4 | 8 (5.41) | 8 (5.44) | 8 (5.48) | 8 (5.26) |  |
| 5 | 8 (5.41) | 8 (5.44) | 8 (5.48) | 8 (5.26) |  |
| 6 | 7 (4.73) | 7 (4.76) | 7 (4.79) | 7 (4.61) |  |
| 7 | 3 (2.03) | 3 (2.04) | 3 (2.05) | 3 (1.97) |  |
| Ca (12 Wks) [Mean (SD)] | 9.19 (0.32) | 9.19 (0.32) | 9.19 (0.32) | 9.21 (0.32) | $N\left(\mu_{k},\sigma_{k}^{2}\right)$ |
| Ca at 28 Wks [Mean (SD)] | 8.83 (0.36) | 8.82 (0.36) | 8.83 (0.36) | 8.83 (0.36) | $N\left(\mu_{k},\sigma_{k}^{2}\right)$ |
| Ca at 36 Wks [Mean (SD)] | 8.89 (0.34) | 8.89 (0.34) | 8.89 (0.34) | 8.91 (0.33) | $N\left(\mu_{k},\sigma_{k}^{2}\right)$ |
| P at 12 Wks [Mean (SD)] | 3.98 (0.53) | 3.97 (0.54) | 3.94 (0.54) | 3.98 (0.53) | $N\left(\mu_{k},\sigma_{k}^{2}\right)$ |
| P at 28 Wks [Mean (SD)] | 3.72 (0.57) | 3.72 (0.57) | 3.72 (0.58) | 3.73 (0.55) | $N\left(\mu_{k},\sigma_{k}^{2}\right)$ |
| P at 36 Wks [Mean (SD)] | 4.01 (0.65) | 4.01 (0.65) | 4.01 (0.66) | 4.01 (0.66) | $N\left(\mu_{k},\sigma_{k}^{2}\right)$ |
| OHD at 12 Wks [Mean (SD)] | 22.49 (10.4) | 22.42 (10.5) | 22.62 (10.4) | 22.58 (10.3) | $N\left(\mu_{k},\sigma_{k}^{2}\right)$ |
| OHD at 28 Wks [Mean (SD)] | 37.66 (14.5) | 37.52 (14.5) | 37.85 (14.5) | 37.27 (14.3) | $N\left(\mu_{k},\sigma_{k}^{2}\right)$ |
| OHD at 36 Wks [Mean (SD)] | 39.94 (16.0) | 39.79 (16.1) | 40.07 (16.0) | 39.39 (16.2) | $N\left(\mu_{k},\sigma_{k}^{2}\right)$ |
| PTH at 12 Wks [Mean (SD)] | 18.91 (10.9) | 18.83 (10.9) | 18.83 (10.8) | 18.17 (10.3) | $N\left(\mu_{k},\sigma_{k}^{2}\right)$ |
| PTH at 28 Wks [Mean (SD)] | 20.34 (11.7) | 20.51 (11.7) | 20.22 (11.8) | 20.19 (11.5) | $N\left(\mu_{k},\sigma_{k}^{2}\right)$ |
| PTH at 36 Wks [Mean (SD)] | 20.30 (13.1) | 20.37 (13.1) | 20.35 (13.1) | 20.19 (12.9) | $N\left(\mu_{k},\sigma_{k}^{2}\right)$ |
| **Child** |  |  |  |  |  |
| Gestational Age [Mean (SD)] | 38.73 (2.26) | 38.73 (2.25) | 38.75 (2.25) | 38.81 (2.24) | $N\left(\mu_{k},\sigma_{k}^{2}\right)$ |
| Formula = Yes (%) | 92 (70.77) | 92 (71.32) | 91 (71.09) | 93 (69.92) |  |
| Ca [Mean (SD)] | 10.04 (0.63) | 10.04 (0.63) | 10.06 (0.62) | 10.06 (0.63) | $N\left(\mu_{k},\sigma_{k}^{2}\right)$ |
| P [Mean (SD)] | 5.59 (1.15) | 5.98 (1.15) | 6.00 (1.15) | 6.02 (1.14) | $N\left(\mu_{k},\sigma_{k}^{2}\right)$ |
| OHD [Mean (SD)] | 22.54 (10.5) | 22.54 (10.5) | 22.64 (10.5) | 22.53 (10.5) | $N\left(\mu_{k},\sigma_{k}^{2}\right)$ |
| PTH [Mean (SD)] | 9.02 (8.63) | 9.08 (8.65) | 9.05 (8.70) | 9.46 (8.78) | $N\left(\mu_{k},\sigma_{k}^{2}\right)$ |
| OH2D [Mean (SD)] | 36.85 (14.3) | 36.72 (14.4) | 36.92 (14.4) | 37.13 (15.0) | $N\left(\mu_{k},\sigma_{k}^{2}\right)$ |
| Vit-D BP [Count (%)] |  |  |  |  | $Multinomial(v_{1k},v_{2k},v_{3k})$ |
| 1 (1F/1F) | 23 (24.47) | 23 (24.73) | 23 (24.47) | 24 (25) |  |
| 2 (1F/2, 1S/1F, 1S/2, 2/2) | 53 (56.38) | 53 (56.99) | 53 (56.38) | 55 (57.29) |  |
| 3 (1S/1S) | 18 (19.15) | 17 (18.28) | 18 (19.15) | 17 (17.71) |  |
| Age at Visit [Mean (SD)] | 3.95 (1.01) | 3.94 (0.99) | 3.97 (1.00) | 3.97 (0.99) | $N\left(\mu_{k},\sigma_{k}^{2}\right)$ |
| DDS = Yes (%) | 107 (80.45) | 106 (80.92) | 107 (81.06) | 112 (83.58) |  |
| Fltx = Yes (%) | 47 (38.21) | 46 (38.02) | 47 (38.52) | 48 (38.71) |  |
| Sex = Male (%) | 77 (52.03) | 76 (51.7) | 76 (52.05) | 78 (51.32) |  |
| Child Strep Mutans [Count (%)] |  |  |  |  | $Poisson\left(\lambda_{k}\right)$ |
| 0 | 100 (68.97) | 99 (68.75) | 99 (69.23) | 100 (67.11) |  |
| 1 | 22 (15.17) | 22 (15.28) | 22 (15.38) | 24 (16.11) |  |
| 2 | 18 (12.41) | 18 (12.5) | 18 (12.59) | 19 (12.75) |  |
| 3 | 5 (3.45) | 5 (3.47) | 4 (2.8) | 6 (4.03) |  |

***Table S 2 – Parameter Magnitudes Set for Each Simulation Scenario (OP)***

|  | **Scenario 1** | | | **Scenario 2** | | | **Scenario 3** | | |
| --- | --- | --- | --- | --- | --- | --- | --- | --- | --- |
|  | Cervical | Middle | Incisal | Cervical | Middle | Incisal | Cervical | Middle | Incisal |
| Intercept | -1.248 | -0.844 | -0.987 | -1.248 | -0.844 | -0.987 | 0.3 | 0.55 | 0.45 |
| Child’s OH2D | 0.007 | -0.001 | -0.02 | 0.007 | -0.001 | -0.02 | 0.95 | 1.05 | 0.75 |
| Child’s Ca | -0.126 | 0.049 | 0.106 | -0.126 | 0.049 | 0.106 | 0.4 | 0.8 | 0.6 |
| Child’s P | -0.006 | -0.24 | -0.026 | -0.006 | -0.24 | -0.026 | 0 | 0 | 0 |
| Gestational Age | 0.062 | 0.106 | 0.674 | 0.062 | 0.106 | 0.674 | 0 | 0 | 0 |
| Formula | -0.169 | -0.306 | -0.413 | -0.169 | -0.306 | -0.413 | 0 | 0 | 0 |
| Mother’s Age | -0.017 | 0.025 | 0.005 | -0.017 | 0.025 | 0.005 | 1.8 | 0.6 | 1.4 |
| Mother’s BMI | 0.028 | 0.066 | -0.043 | 0.028 | 0.066 | -0.043 | 0 | 0 | 0 |
| Antacid Counts | 0.007 | -0.222 | -0.025 | 0.007 | -0.222 | -0.025 | 0.85 | 0.7 | 0.9 |
| Mother’s Ca at 12 Wks | -0.073 | -0.159 | -0.024 | -0.073 | -0.159 | -0.024 | 0 | 0 | 0 |
| Mother’s Ca at 28 Wks | 0.043 | -0.458 | 0.023 | 0.043 | -0.458 | 0.023 | 0 | 0 | 0 |
| Mother’s Ca at 36 Wks | -0.147 | 0.143 | 0.093 | -0.147 | 0.143 | 0.093 | 0 | 0 | 0 |
| Mother’s P at 12 Wks | 0.184 | 0.005 | -0.143 | 0.184 | 0.005 | -0.143 | 0 | 0 | 0 |
| Mother’s P at 28 Wks | 0.145 | -0.034 | -0.029 | 0.145 | -0.034 | -0.029 | 0.6 | 0.4 | 0.8 |
| Mother’s P at 36 Wks | 0.037 | 0.248 | 0.037 | 0.037 | 0.248 | 0.037 | 0 | 0 | 0 |
| Child’s FVDD | 0.001 | -0.137 | 0.016 | 0.001 | -0.137 | 0.016 | 0 | 0 | 0 |
| Mother’s FVDD at 12 Wks | -0.334 | -0.051 | -0.102 | -0.334 | -0.051 | -0.102 | 0.45 | 0.75 | 0.65 |
| Mother’s FVDD at 28 Wks | -0.017 | 0.004 | 0.025 | -0.017 | 0.004 | 0.025 | 0 | 0 | 0 |
| Mother’s FVDD at 36 Wks | 0.004 | -0.046 | -0.334 | 0.004 | -0.046 | -0.334 | 0 | 0 | 0 |
| Vit-D (Cat. 1) | 0.164 | -0.301 | -0.172 | 0.164 | -0.301 | -0.172 | 1.1 | 0.9 | 1.3 |
| Vit-D (Cat. 2) | -0.181 | -0.15 | -0.1 | -0.181 | -0.15 | -0.1 | 1.5 | 1.4 | 1.2 |
| Child’s Age at Visit | 0.048 | 0.162 | 0.021 | 0.048 | 0.162 | 0.021 | 0 | 0 | 0 |
| Child’s DDS | -0.886 | -0.287 | -0.187 | -0.886 | -0.287 | -0.187 | 0 | 0 | 0 |
| Child’s Fltx | -0.108 | -0.082 | -0.09 | -0.108 | -0.082 | -0.09 | 0.9 | 1 | 0.5 |
| Child’s Sex | -0.117 | -0.018 | -0.725 | -0.117 | -0.018 | -0.725 | 0 | 0 | 0 |
| Child’s Strep Mutans Cnt | 0.229 | 0.131 | -0.353 | 0.229 | 0.131 | -0.353 | 1 | 2 | 1.5 |

*Table S2 Description: Parameter magnitudes used for Scenarios 1 and 2 are based on the posterior means of the parameters from original application model for the opacity defect. Parameter magnitudes for Scenario 3 are randomly assigned with the number of predictors chosen based on the number of predictors that met the threshold for inclusion in the original application model for opacity.*

***Table S 3 - Magnitudes Used for Covariance Structure (OP)***

|  | **Scenario 1** | | | **Scenario 2** | | | **Scenario 3** | | |
| --- | --- | --- | --- | --- | --- | --- | --- | --- | --- |
|  | Cervical | Middle | Incisal | Cervical | Middle | Incisal | Cervical | Middle | Incisal |
| Cervical | 1.0000 | 0.1359 | -0.0875 | 1.0000 | 0.0000 | 0.0000 | 1.0000 | 0.1359 | -0.0875 |
| Middle | 0.1359 | 0.8524 | 0.3521 | 0.0000 | 1.0000 | 0.0000 | 0.1359 | 0.8524 | 0.3521 |
| Incisal | -0.0875 | 0.3521 | 0.6194 | 0.0000 | 0.0000 | 1.0000 | -0.0875 | 0.3521 | 0.6194 |

*Table S3 Description: Estimates for the covariance matrix obtained for Scenarios 1 and 3 are based on the posterior means of the LKJ covariance structure from original application model for the opacity defect. Estimates for the covariance matrix for Scenario 2 are under the assumption that the regions are independent.*

***Table S 4 - Parameter Magnitudes Set for Each Simulation Scenario (PEB)***

|  | **Scenario 1** | **Scenario 2** | **Scenario 3** |
| --- | --- | --- | --- |
|  | Incisal | Incisal | Incisal |
| Intercept | 0.071 | 0.071 | 0.5 |
| Child’s OH2D | 0.03 | 0.03 | 0 |
| Child’s Ca | -0.204 | -0.204 | 0 |
| Child’s P | -0.261 | -0.261 | 0 |
| Gestational Age | -0.078 | -0.078 | 0 |
| Formula | 0.03 | 0.03 | 0 |
| Mother’s Age | -0.005 | -0.005 | 1.8 |
| Mother’s BMI | -1.058 | -1.058 | 0 |
| Antacid Counts | -0.026 | -0.026 | 0 |
| Mother’s Ca at 12 Wks | -0.077 | -0.077 | 0 |
| Mother’s Ca at 28 Wks | 0.027 | 0.027 | 0 |
| Mother’s Ca at 36 Wks | -0.083 | -0.083 | 0 |
| Mother’s P at 12 Wks | 0.014 | 0.014 | 0 |
| Mother’s P at 28 Wks | 0.49 | 0.49 | 0.6 |
| Mother’s P at 36 Wks | -0.001 | -0.001 | 0 |
| Child’s FVDD | -0.009 | -0.009 | 0 |
| Mother’s FVDD at 12 Wks | -0.02 | -0.02 | 0.45 |
| Mother’s FVDD at 28 Wks | -0.032 | -0.032 | 0 |
| Mother’s FVDD at 36 Wks | -0.016 | -0.016 | 0 |
| Vit-D (Cat. 1) | -0.017 | -0.017 | 1.1 |
| Vit-D (Cat. 2) | -0.137 | -0.137 | 0 |
| Child’s Age at Visit | 0.955 | 0.955 | 0 |
| Child’s DDS | 0.002 | 0.002 | 0 |
| Child’s Fltx | 0.069 | 0.069 | 0 |
| Child’s Sex | -0.075 | -0.075 | 0 |
| Child’s Strep Mutans Count | 0.049 | 0.049 | 0 |

*Table S4 Description: Parameter magnitudes used for Scenarios 1 and 2 are based on the posterior means of the parameters from original application model for the post-eruptive breakdown defect. Parameter magnitudes for Scenario 3 are randomly assigned with the number of predictors chosen based on the number of predictors that met the threshold for inclusion in the original application model for post-eruptive breakdown.*

***Table S 5 - Parameter Magnitudes Set for Each Simulation Scenario (DC)***

|  | **Scenario 1** | | | **Scenario 2** | | | **Scenario 3** | | |
| --- | --- | --- | --- | --- | --- | --- | --- | --- | --- |
|  | Cervical | Middle | Incisal | Cervical | Middle | Incisal | Cervical | Middle | Incisal |
| Intercept | -0.114 | -0.776 | -0.971 | -0.114 | -0.776 | -0.971 | 0.5 | 0.25 | 0.75 |
| Child’s OH2D | 0.01 | 0.005 | 0.018 | 0.01 | 0.005 | 0.018 | 0 | 0 | 0 |
| Child’s Ca | -0.19 | 0.241 | 0.046 | -0.19 | 0.241 | 0.046 | 0.4 | 0.8 | 0.6 |
| Child’s P | 0.134 | 0.05 | 0.031 | 0.134 | 0.05 | 0.031 | 0 | 0 | 0 |
| Gestational Age | 0.123 | 0.009 | -0.085 | 0.123 | 0.009 | -0.085 | 0 | 0 | 0 |
| Formula | -0.082 | -0.275 | -0.22 | -0.082 | -0.275 | -0.22 | 0 | 0 | 0 |
| Mother’s Age | 0.02 | 0.006 | 0.019 | 0.02 | 0.006 | 0.019 | 1.8 | 0.6 | 1.4 |
| Mother’s BMI | -0.116 | 0.001 | -0.016 | -0.116 | 0.001 | -0.016 | 0 | 0 | 0 |
| Antacid Counts | -0.186 | -0.448 | -0.531 | -0.186 | -0.448 | -0.531 | 0 | 0 | 0 |
| Mother’s Ca at 12 Wks | 0.109 | 0.148 | -0.125 | 0.109 | 0.148 | -0.125 | 0 | 0 | 0 |
| Mother’s Ca at 28 Wks | 0.079 | 0.12 | -0.119 | 0.079 | 0.12 | -0.119 | 0 | 0 | 0 |
| Mother’s Ca at 36 Wks | 0.026 | 0.105 | -0.03 | 0.026 | 0.105 | -0.03 | 0 | 0 | 0 |
| Mother’s P at 12 Wks | 0.032 | -0.179 | 0.1 | 0.032 | -0.179 | 0.1 | 0 | 0 | 0 |
| Mother’s P at 28 Wks | 0.073 | 0.158 | -0.04 | 0.073 | 0.158 | -0.04 | 0 | 0 | 0 |
| Mother’s P at 36 Wks | -0.065 | 0.177 | -0.058 | -0.065 | 0.177 | -0.058 | 0 | 0 | 0 |
| Child’s FVDD | 0.006 | -0.044 | -0.062 | 0.006 | -0.044 | -0.062 | 0 | 0 | 0 |
| Mother’s FVDD at 12 Wks | -0.168 | -0.081 | -0.078 | -0.168 | -0.081 | -0.078 | 0 | 0 | 0 |
| Mother’s FVDD at 28 Wks | -0.115 | -0.127 | -0.284 | -0.115 | -0.127 | -0.284 | 0 | 0 | 0 |
| Mother’s FVDD at 36 Wks | -0.901 | -0.993 | -0.794 | -0.901 | -0.993 | -0.794 | 0 | 0 | 0 |
| Vit-D (Cat. 1) | 0.04 | -0.108 | -0.371 | 0.04 | -0.108 | -0.371 | 1.1 | 0.9 | 1.3 |
| Vit-D (Cat. 2) | -0.153 | -0.265 | 0.048 | -0.153 | -0.265 | 0.048 | 0 | 0 | 0 |
| Child’s Age at Visit | -0.235 | 0.182 | 0.271 | -0.235 | 0.182 | 0.271 | 0 | 0 | 0 |
| Child’s DDS | -0.192 | -0.278 | -0.076 | -0.192 | -0.278 | -0.076 | 0 | 0 | 0 |
| Child’s Fltx | -0.031 | -0.04 | -0.169 | -0.031 | -0.04 | -0.169 | 0 | 0 | 0 |
| Child’s Sex | -0.074 | -0.067 | -0.18 | -0.074 | -0.067 | -0.18 | 0 | 0 | 0 |
| Child’s Strep Mutans Cnt | 0.407 | 0.142 | -0.272 | 0.407 | 0.142 | -0.272 | 1 | 2 | 1.5 |

*Table S5 Description: Parameter magnitudes used for Scenarios 1 and 2 are based on the posterior means of the parameters from original application model for the dental caries defect. Parameter magnitudes for Scenario 3 are randomly assigned with the number of predictors chosen based on the number of predictors that met the threshold for inclusion in the original application model for dental caries.*

***Table S 6 - Magnitudes Used for Covariance Structure (DC)***

|  | **Scenario 1** | | | **Scenario 2** | | | **Scenario 3** | | |
| --- | --- | --- | --- | --- | --- | --- | --- | --- | --- |
|  | Cervical | Middle | Incisal | Cervical | Middle | Incisal | Cervical | Middle | Incisal |
| Cervical | 1.0000 | 0.1804 | 0.0470 | 1.0000 | 0.0000 | 0.0000 | 1.0000 | 0.1804 | 0.0470 |
| Middle | 0.1804 | 0.8395 | 0.0181 | 0.0000 | 1.0000 | 0.0000 | 0.1804 | 0.8395 | 0.0181 |
| Incisal | 0.0470 | 0.0181 | 0.6819 | 0.0000 | 0.0000 | 1.0000 | 0.0470 | 0.0181 | 0.6819 |

*Table S6 Description: Estimates for the covariance matrix obtained for Scenarios 1 and 3 are based on the posterior means of the LKJ covariance structure from original application model for the dental caries defect. Estimates for the covariance matrix for Scenario 2 are under the assumption that the regions are independent.*

*Scenario 1: Re-fitting Original Application Model*

***Figure S 1 - Posterior Parameter Means Given Inclusion (EH)***


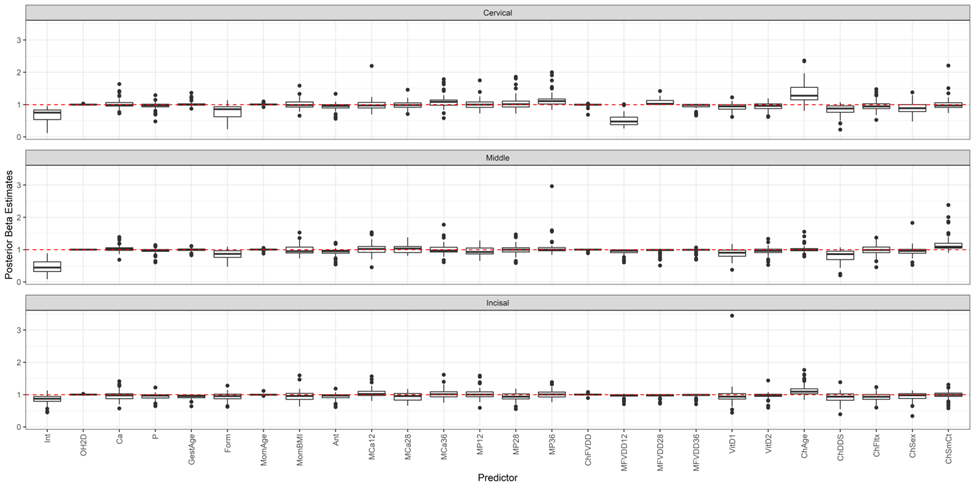


***Description****: Figure S1 details the range of posterior parameter estimates (on the odds scale) given their inclusion for all predictors in the full model. The horizontal red dashed line denotes the null odds of 1.*

***Figure S 2 - MAE in Posterior Parameter Estimates Given Inclusion (EH)***

*

*

***Description****: Figure S2 details the mean absolute error between the original application’s posterior parameter means and the posterior means obtained from the iterations of the simulation given their inclusion.*

***Figure S 3 - Posterior Probabilities of Inclusion (OP)***





***Description****: Figure S3 details the median and range of the posterior probabilities of inclusion across the 50 iterations of the simulation for opacity. The horizontal red dashed line denotes the stricter threshold of inclusion (set at 0.6) based on the large number of predictors in our model. The horizontal blue dashed line denotes the original threshold of inclusion at 0.5. The red points shown in each box plot are the full models’ posterior probability of inclusion from the original application for that predictor in that region.*

***Figure S 4 - Posterior Parameter Means Given Inclusion (OP)***





***Description****: Figure S4 details the range of posterior parameter estimates (on the odds scale) for opacity given their inclusion for all predictors in the full model. The horizontal red dashed line denotes the null odds of 1.*

***Figure S 5 - MSE in Posterior Parameter Estimates Given Inclusion (OP)***

*

*

***Description****: Figure S5 details the mean-squared error between the original application’s posterior parameter means and the posterior means obtained from the iterations of the simulation given their inclusion for opacity.*

***Figure S 6 - MAE in Posterior Parameter Estimates Given Inclusion (OP)***

*

*

***Description****: Figure S6 details the mean absolute error between the original application’s posterior parameter means and the posterior means obtained from the iterations of the simulation given their inclusion for opacity.*

***Figure S 7 - Posterior Probabilities of Inclusion (PEB)***





***Description****: Figure S7 details the median and range of the posterior probabilities of inclusion across the 50 iterations of the simulation for post-eruptive breakdown. The horizontal red dashed line denotes the stricter threshold of inclusion (set at 0.6) based on the large number of predictors in our model. The horizontal blue dashed line denotes the original threshold of inclusion at 0.5. The red points shown in each box plot are the full models’ posterior probability of inclusion from the original application for that predictor in that region.*

***Figure S 8 - Posterior Parameter Means Given Inclusion (PEB)***





***Description****: Figure S8 details the range of posterior parameter estimates (on the odds scale) for post-eruptive breakdown given their inclusion for all predictors in the full model. The horizontal red dashed line denotes the null odds of 1.*

***Figure S 9 - MSE in Posterior Parameter Estimates Given Inclusion (PEB)***

*

*

***Description****: Figure S9 details the mean-squared error between the original application’s posterior parameter means and the posterior means obtained from the iterations of the simulation given their inclusion for post-eruptive breakdown.*

***Figure S 10 - MAE in Posterior Parameter Estimates Given Inclusion (PEB)***

*

*

***Description****: Figure S10 details the mean absolute error between the original application’s posterior parameter means and the posterior means obtained from the iterations of the simulation given their inclusion for post-eruptive breakdown.*

***Figure S 11 - Posterior Probabilities of Inclusion (DC)***





***Description****: Figure S11 details the median and range of the posterior probabilities of inclusion across the 50 iterations of the simulation for dental caries. The horizontal red dashed line denotes the stricter threshold of inclusion (set at 0.6) based on the large number of predictors in our model. The horizontal blue dashed line denotes the original threshold of inclusion at 0.5. The red points shown in each box plot are the full models’ posterior probability of inclusion from the original application for that predictor in that region.*

***Figure S 12 - Posterior Parameter Means Given Inclusion (DC)***





***Description****: Figure S12 details the range of posterior parameter estimates (on the odds scale) for dental caries given their inclusion for all predictors in the full model. The horizontal red dashed line denotes the null odds of 1.*

***Figure S 13 - MSE in Posterior Parameter Estimates Given Inclusion (DC)***

*

*

***Description****: Figure S13 details the mean-squared error between the original application’s posterior parameter means and the posterior means obtained from the iterations of the simulation given their inclusion for dental caries.*

***Figure S 14 - MAE in Posterior Parameter Estimates Given Inclusion (DC)***

*

*

***Description****: Figure S14 details the mean absolute error between the original application’s posterior parameter means and the posterior means obtained from the iterations of the simulation given their inclusion for dental caries.*

*Scenario 2: Independent Regions*

***Figure S 15 - Posterior Probabilities of Inclusion (EH)***





***Description****: Figure S15 details the median and range of the posterior probabilities of inclusion across the 50 iterations of the simulation for enamel hypoplasia. The horizontal red dashed line denotes the stricter threshold of inclusion (set at 0.6) based on the large number of predictors in our model. The horizontal blue dashed line denotes the original threshold of inclusion at 0.5. The red points shown in each box plot are the full models’ posterior probability of inclusion from the original application for that predictor in that region.*

***Figure S 16 - Posterior Parameter Means Given Inclusion (EH)***





***Description****: Figure S16 details the range of posterior parameter estimates (on the odds scale) given their inclusion for all predictors in the full model. The horizontal red dashed line denotes the null odds of 1.*

***Figure S 17 - MAE in Posterior Covariance Means (EH)***

*

*

***Description****: Figure S17 details the mean absolute error between the original application’s posterior means of the covariance structure for the LKJ correlation and the posterior means of the covariance structure obtained from the iterations of the simulation for enamel hypoplasia.*

***Figure S 18 - Posterior Probabilities of Inclusion (OP)***





***Description****: Figure S18 details the median and range of the posterior probabilities of inclusion across the 50 iterations of the simulation for opacity. The horizontal red dashed line denotes the stricter threshold of inclusion (set at 0.6) based on the large number of predictors in our model. The horizontal blue dashed line denotes the original threshold of inclusion at 0.5. The red points shown in each box plot are the full models’ posterior probability of inclusion from the original application for that predictor in that region.*

***Figure S 19 - Posterior Parameter Means Given Inclusion (OP)***





***Description****: Figure S19 details the range of posterior parameter estimates (on the odds scale) for opacity given their inclusion for all predictors in the full model. The horizontal red dashed line denotes the null odds of 1.*

***Figure S 20 - MSE in Posterior Covariance Means (OP)***

*

*

***Description****: Figure S20 details the mean-squared error between the original application’s posterior means of the covariance structure for the LKJ correlation and the posterior means of the covariance structure obtained from the iterations of the simulation for opacity.*

***Figure S 21 - MAE in Posterior Covariance Means (OP)***

*

*

***Description****: Figure S21 details the mean absolute error between the original application’s posterior means of the covariance structure for the LKJ correlation and the posterior means of the covariance structure obtained from the iterations of the simulation for opacity.*

***Figure S 22 - Posterior Probabilities of Inclusion (DC)***





***Description****: Figure S22 details the median and range of the posterior probabilities of inclusion across the 50 iterations of the simulation for dental caries. The horizontal red dashed line denotes the stricter threshold of inclusion (set at 0.6) based on the large number of predictors in our model. The horizontal blue dashed line denotes the original threshold of inclusion at 0.5. The red points shown in each box plot are the full models’ posterior probability of inclusion from the original application for that predictor in that region.*

***Figure S 23 - Posterior Parameter Means Given Inclusion (DC)***





***Description****: Figure S23 details the range of posterior parameter estimates (on the odds scale) for dental caries given their inclusion for all predictors in the full model. The horizontal red dashed line denotes the null odds of 1.*

***Figure S 24 - MSE in Posterior Covariance Means (DC)***

*

*

***Description****: Figure S24 details the mean-squared error* *between the original application’s posterior means of the covariance structure for the LKJ correlation and the posterior means of the covariance structure obtained from the iterations of the simulation for dental caries.*

***Figure S 25 - MAE in Posterior Covariance Means (DC)***

*

*

***Description****: Figure S25 details the mean absolute error* *between the original application’s posterior means of the covariance structure for the LKJ correlation and the posterior means of the covariance structure obtained from the iterations of the simulation for dental caries.*

*Scenario 3: Adjusting Posterior Means*

***Figure S 26 – Posterior Parameter Means Given Inclusion (EH)***





***Description****: Figure S26 details the range of posterior parameter estimates (on the odds scale) given their inclusion for all predictors in the full model. The horizontal red dashed line denotes the null odds of 1.*

***Figure S 27 - MSE in Posterior Parameter Estimates Given Inclusion (EH)***

*

*

***Description****: Figure S27 details the mean-squared error between the set posterior parameter means and the posterior means obtained from the iterations of the simulation given their inclusion.*

***Figure S 28 - MAE in Posterior Parameter Estimates Given Inclusion (EH)***

*

*

***Description****: Figure S28 details the mean absolute error between the set posterior parameter means and the posterior means obtained from the iterations of the simulation given their inclusion.*

***Figure S 29 - Posterior Probabilities of Inclusion (OP)***





***Description****: Figure S29 details the median and range of the posterior probabilities of inclusion across the 50 iterations of the simulation for opacity. The horizontal red dashed line denotes the stricter threshold of inclusion (set at 0.6) based on the large number of predictors in our model. The horizontal blue dashed line denotes the original threshold of inclusion at 0.5.*

***Figure S 30 - Posterior Parameter Means Given Inclusion (OP)***





***Description****: Figure S30 details the range of posterior parameter estimates (on the odds scale) for opacity given their inclusion for all predictors in the full model. The horizontal red dashed line denotes the null odds of 1.*

***Figure S 31 - MSE in Posterior Parameter Estimates Given Inclusion (OP)***

*

*

***Description****: Figure S31 details the mean-squared error between the set posterior means and the posterior parameter means obtained from the iterations of the simulation given their inclusion for opacity.*

***Figure S 32 - MAE in Posterior Parameter Estimates Given Inclusion (OP)***

*

*

***Description****: Figure S32 details the mean absolute error between the set posterior parameter means and the posterior means obtained from the iterations of the simulation given their inclusion for opacity.*

***Figure S 33 - Posterior Probabilities of Inclusion (PEB)***





***Description****: Figure S33 details the median and range of the posterior probabilities of inclusion across the 50 iterations of the simulation for post-eruptive breakdown. The horizontal red dashed line denotes the stricter threshold of inclusion (set at 0.6) based on the large number of predictors in our model. The horizontal blue dashed line denotes the original threshold of inclusion at 0.5.*

***Figure S 34 - Posterior Parameter Means Given Inclusion (PEB)***





***Description****: Figure S34 details the range of posterior parameter estimates (on the odds scale) for post-eruptive breakdown given their inclusion for all predictors in the full model. The horizontal red dashed line denotes the null odds of 1.*

***Figure S 35 - MSE in Posterior Parameter Estimates Given Inclusion (PEB)***

*

*

***Description****: Figure S35 details the mean-squared error between the set posterior parameter means and the posterior means obtained from the iterations of the simulation given their inclusion for post-eruptive breakdown.*

***Figure S 36 - MAE in Posterior Parameter Estimates Given Inclusion (PEB)***

*

*

***Description****: Figure S36 details the mean absolute error between the set posterior parameter means and the posterior means obtained from the iterations of the simulation given their inclusion for post-eruptive breakdown.*

***Figure S 37 - Posterior Probabilities of Inclusion (DC)***





***Description****: Figure S37 details the median and range of the posterior probabilities of inclusion across the 50 iterations of the simulation for dental caries. The horizontal red dashed line denotes the stricter threshold of inclusion (set at 0.6) based on the large number of predictors in our model. The horizontal blue dashed line denotes the original threshold of inclusion at 0.5.*

***Figure S 38 - Posterior Parameter Means Given Inclusion (DC)***





***Description****: Figure S38 details the range of posterior parameter estimates (on the odds scale) for dental caries given their inclusion for all predictors in the full model. The horizontal red dashed line denotes the null odds of 1.*

***Figure S 39 - MSE in Posterior Parameter Estimates Given Inclusion (DC)***

*

*

***Description****: Figure S39 details the mean-squared error between the set posterior parameter means and the posterior means obtained from the iterations of the simulation given their inclusion for dental caries.*

***Figure S 40 - MAE in Posterior Parameter Estimates Given Inclusion (DC)***

*

*

***Description****: Figure S40 details the mean absolute error between the set posterior parameter means and the posterior means obtained from the iterations of the simulation given their inclusion for dental caries.*
